# Effects of cortisol on female-to-male sex change in a wrasse

**DOI:** 10.1371/journal.pone.0273779

**Published:** 2022-09-01

**Authors:** Alexander Goikoetxea, Erica V. Todd, Simon Muncaster, P. Mark Lokman, Jodi T. Thomas, Holly A. Robertson, Carlos E. De Farias e Moraes, Neil J. Gemmell

**Affiliations:** 1 Department of Anatomy, School of Biomedical Sciences, University of Otago, Dunedin, New Zealand; 2 School of Life and Environmental Sciences, Deakin University, Geelong, Australia; 3 Environmental Management Group, Toi Ohomai Institute of Technology, Tauranga, New Zealand; 4 School of Science, University of Waikato, Tauranga, New Zealand; 5 Department of Zoology, University of Otago, Dunedin, New Zealand; National Cheng Kung University, TAIWAN

## Abstract

Sex change occurs as a usual part of the life cycle for many teleost fish and the modifications involved (behavioural, gonadal, morphological) are well studied. However, the mechanism that transduces environmental cues into the molecular cascade that underlies this transformation remains unknown. Cortisol, the main stress hormone in fish, is hypothesised to be a key factor linking environmental stimuli with sex change by initiating gene expression changes that shift steroidogenesis from oestrogens to androgens but this notion remains to be rigorously tested. Therefore, this study aimed to experimentally test the role of cortisol as an initiator of sex change in a protogynous (female-to-male) hermaphrodite, the New Zealand spotty wrasse (*Notolabrus celidotus*). We also sought to identify potential key regulatory factors within the head kidney that may contribute to the initiation and progression of gonadal sex change. Cortisol pellets were implanted into female spotty wrasses under inhibitory conditions (presence of a male), and outside of the optimal season for natural sex change. Histological analysis of the gonads and sex hormone analyses found no evidence of sex change after 71 days of cortisol treatment. However, expression analyses of sex and stress-associated genes in gonad and head kidney suggested that cortisol administration did have a physiological effect. In the gonad, this included upregulation of *amh*, a potent masculinising factor, and *nr3c1*, a glucocorticoid receptor. In the head kidney, *hsd11b2*, which converts cortisol to inactive cortisone to maintain cortisol balance, was upregulated. Overall, our results suggest cortisol administration outside of the optimal sex change window is unable to initiate gonadal restructuring. However, our expression data imply key sex and stress genes are sensitive to cortisol. This includes genes expressed in both gonad and head kidney that have been previously implicated in early sex change in several sex-changing species.

## Introduction

Sex change occurs as a usual part of the life cycle for many teleost fishes [1], is often socially cued, and involves dramatic changes in behaviour, gonadal architecture, and gene expression [2–4]. Changing sex can enhance the lifetime reproductive fitness of individuals [5]. However, how sex change occurs remains largely unknown [6]. It seems clear that the process of sex change begins in the brain [7], but exactly how visual cues are perceived and translated into fast and potent behavioural responses and subsequent gonadal sex change remains to be fully characterised [8,9].

Multiple lines of evidence strongly implicate the interaction between the hypothalamic-pituitary-interrenal (HPI) and -gonadal (HPG) axes in the sex change process of teleost hermaphroditic fish [10]. Fluctuations (e.g., hormonal) regulated by the HPG axis are crucial during sexual development and the control of reproduction. Meanwhile, an environmental stressor (e.g., increased temperature, social cues) can activate the HPI axis (also known as the stress axis and equivalent to the vertebrate HPA (hypothalamic-pituitary-adrenal) axis). Cortisol is the main glucocorticoid in teleost fish and the hormone most directly associated with stress [11,12, reviewed by 13]. Cortisol has emerged as a potential key mediator of sex determination, linking changes in the social environment with the trigger of sex reversal [14,15]. Cortisol treatment promoting masculinisation of genetic females has been described in several gonochoristic fishes (fixed separate sexes), such as the Japanese flounder (*Paralichthys olivaceus*), Southern flounder (*Paralichthys lethostigma*), pejerrey (*Odontesthes bonariensis*) and medaka (*Oryzias latipes*) [16–20]. In naturally hermaphroditic species too, the masculinising effects of cortisol have been documented during sex change in the protogynous three-spot wrasse (*Halichoeres trimaculatus*) [15] and the sandperch (*Parapercis cylindrica*) [21], and in the masculinisation of sexually undifferentiated black sea bass (*Centropristis striata*) [22].

However, in other studies the role for cortisol in sex change is less clearly established. For example, while serum cortisol levels were observed to increase during sex change in the protandrous (male-to-female) cinnamon clownfish (*Amphiprion melanopus*), this rise took place in the late stages of the transformation [23], implying such variation may have been a consequence rather than a cause of sex change. This view is further supported by recent findings in the gonochoristic European sea bass (*Dicentrarchus labrax*), in which natural cortisol fluctuations were found to be independent from sex determination and differentiation mechanisms [24,25]. Such discoveries also agree with contrasting observations in reptilian systems, in which the effect of increased yolk corticosterone influenced sex ratios in some lizard species [26], but not others [27,28]. Thus, cortisol’s role in sex determination may be species-specific.

Where cortisol exerts an effect on sex determinisation and differentiation it may do so by influencing sex-specific gene expression in favour of androgen production and apoptosis in the gonad, thereby promoting maleness [reviewed by 29]. In protogynous hermaphroditic fish, a recent hypothesis suggests that cortisol, together with epigenetic (e.g., DNA-methylation) and genetic factors (e.g., *amh* upregulation), could downregulate the expression of gonadal aromatase (*cyp19a1a*), which converts testosterone (T) to 17β-oestradiol (E2), to trigger sex change [6,28–33]. The effect of cortisol on the promotion or inhibition of aromatase activity [18,20,34,35] or 11-ketotestosterone (11KT) production [20,36–38] in the gonad of gonochoristic fishes has also gained some attention. While changes in the expression of aromatase and other genes in both the gonad and brain have been identified as critical events in protogynous sex change [39,40], the role of the head kidney, the key site of cortisol synthesis, in sex change has not yet been investigated. Likewise, the steroidogenic capacity of the head kidney has been experimentally demonstrated in rainbow trout (*Oncorhynchus mykiss*), Atlantic salmon (*Salmo salar*), African catfish (*Clarias gariepinus*) and European eel (*Anguilla anguilla*) [41–44], but the role of the head kidney in sex change has not been explicitly explored. Studies on hermaphroditic species that include analyses of processes in both gonad and head kidney are needed to shed light on the potential upstream actions of cortisol on gonadal sex steroid production [45].

The aim of this study was to evaluate the role of cortisol as a trigger of sex change in the protogynous New Zealand spotty wrasse or paketi (*Notolabrus celidotus*). Spotty wrasses are endemic to New Zealand and are the country’s most common wrasse species [46]. They are protogynous hermaphrodites that display socially controlled sex change [46,47]. As observed in most wrasses, spotty wrasses present two colour morphs, an initial phase (IP) and a terminal phase (TP) [46,47]. They are also diandric (two male phenotypes), with IP sneaker males which mimic female behaviour, and dominant TP males which establish breeding territories [46]. IP males and IP females cannot be distinguished by their external morphology [47]. Sex change can be reliably induced in spotty wrasse females by the removal of dominant TP males from established social groups in captivity [48–50] (Muncaster et al., submitted).

Cortisol administration to spotty wrasses reared in laboratory conditions was used to elucidate the potential role of this glucocorticoid in the induction of female-to-male sex change under socially non-permissive conditions (i.e., the presence of a TP male). It was hypothesised that cortisol could override social inhibition and induce sex change in treated females. NanoString digital gene expression technology was used to evaluate the expression of female-promoting and male-promoting gene networks plus key genes involved in cortisol synthesis and metabolism, and epigenetic regulation across sex change in both the level of the gonads and of the head kidney. To the best of our knowledge, this is the first work to evaluate the expression of candidate genes in the head kidney in relation to teleost sex change.

## Materials & methods

### Experimental set-up

The effect of stress as a potential trigger for sex change in spotty wrasse was investigated by implanting cortisol pellets into IP spotty wrasse individuals under socially inhibitory conditions between the months of June–September 2017. This is during the breeding season, which lasts from late July until November, but outside of the optimal window for natural sex change, which occurs between November–May (i.e., outside the breeding season) [49]. Fish were captured around high tide by hook and line off the coast of Tauranga, Bay of Plenty, New Zealand (37.6878° S, 176.1651° E) and kept at the Aquaculture Centre at Toi Ohomai Institute of Technology, Tauranga. Forty-eight IP fish ranging from 108–230 mm total length (TL) and 57.20 g of mean weight (MW) were equally distributed across six 400-litre recirculating seawater systems under a natural photoperiod. Natural sex change was prevented by placing one TP male (115–226 mm TL, 142.47 g MW) in each tank. Fish were fed frozen green-lipped mussels (*Perna canaliculus*) three times a week for the duration of the experiment (ten weeks) (standard practice for this species). The duration of the experiment and the sampling times were estimated based on previous data on the completion of sex change in captive spotty wrasse using an aromatase inhibitor (60 days) or social inhibition (66 days) [49].

Pellets containing 5 mg of cortisol (Sigma-Aldrich) in a matrix of 95 cholesterol: 5 cellulose were made in-house (as described in Sherwood et al. [51] and Lokman et al. [52]). Sham pellets contained matrix only. Prior to the experiment, the release rate of cortisol from pellets was tested both *in vitro* and *in vivo* (pilot study) before experimentation (see Supplemental Materials and Methods). Pellets released most of their cortisol during the first 8–10 days post-implantation.

**Fig 1:**
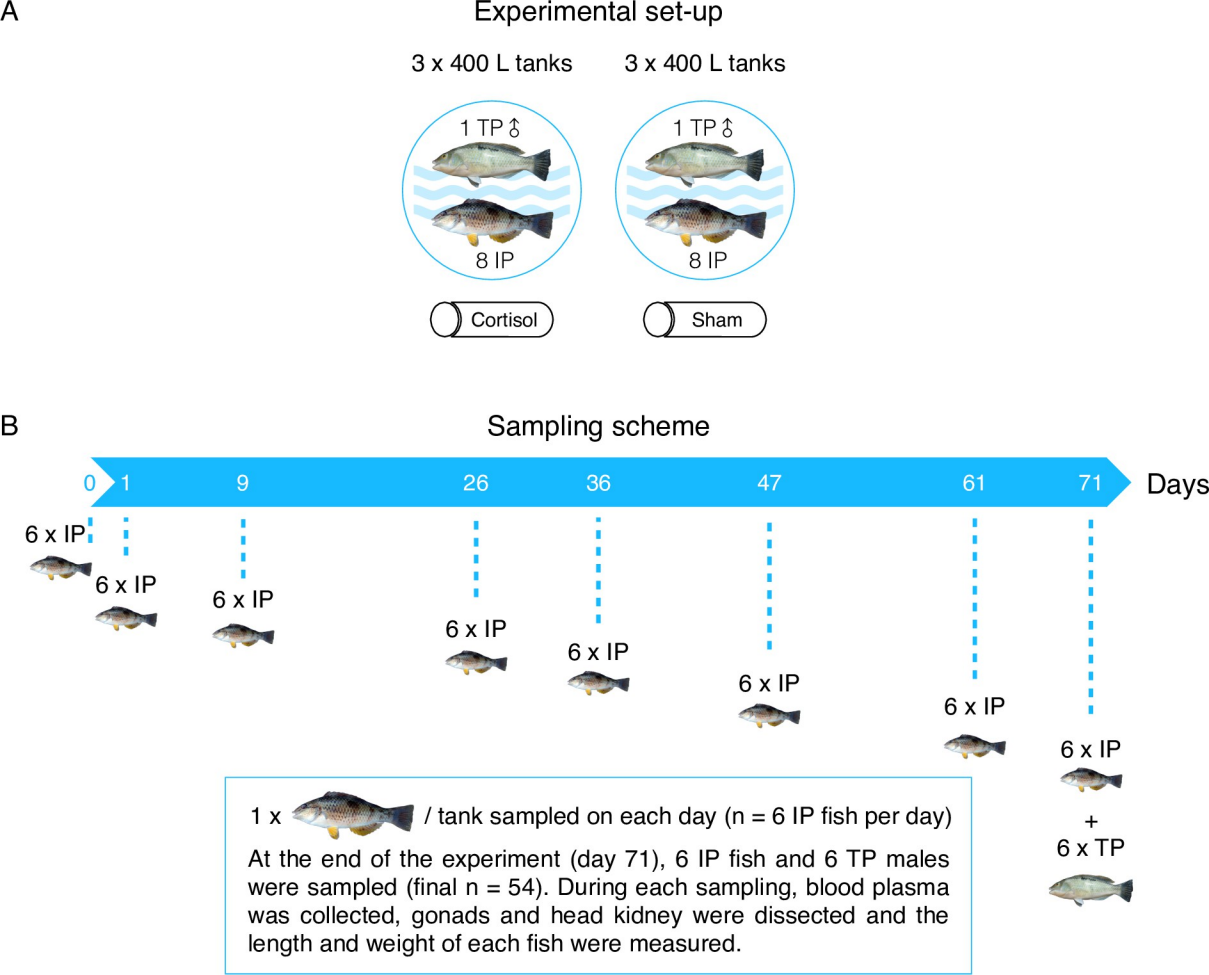
Experimental set-up (A) and sampling scheme (B). Three tanks acted as cortisol-implanted tanks and three as a control throughout the experiment. Tissue samples were collected from one IP fish per tank on days 0, 1, 9, 26, 36, 47, 61 and 71 (end of experiment). During each sampling day, anaesthetic administration, blood plasma collection, tissue dissection and recording of morphometrics were conducted as described in the previous paragraph. IP New Zealand spotty wrasse image by Allan Burgess, TP New Zealand spotty wrasse image by Jodi Thomas.

Following an acclimation period of three weeks, on day 0 of the experiment (late June, before the onset of the breeding season), all fish in each tank were captured simultaneously and anesthetised in an aerated bath containing 600 ppm 2-phenoxyethanol (Sigma-Aldrich). Spotty wrasse were marked with colour T-bar anchor tags (Hallprint) and were given a single intramuscular 5 mg cortisol (treatment) or sham (control) pellet (see Supplemental Materials and Methods) using a Ralgun implanter. One randomly selected IP individual per tank was euthanised for blood and tissue collection to obtain reference steroid and gene expression measures prior to implantation of pellets (day 0 control fish); blood samples were collected using a 1 mL heparinised syringe from the caudal vein, and then fish were euthanised by rapid decapitation. Body weight and length, and gonadal weight were measured for each individual. Tissue samples were always taken from the same region, approximately the middle third of one gonad, for standardisation of histological analysis. Gonad tissue was fixed in 4% PFA (paraformaldehyde) overnight before being transferred to 70% ethanol for histological processing. The second gonad and the head kidney were flash-frozen in isopentane (C_5_H_12_) (Sigma-Aldrich) (kept ice-cold on dry ice) and stored at -80°C until RNA extraction (Fig 1).

Capture and handling may induce an acute stress response, making the measurement of cortisol in plasma unreliable. There is considered to be no detectable effect of handling on cortisol levels when blood samples are taken within three minutes of fish capture [53–55]. However, all of our blood samples were taken later than three minutes after capture (6’ 0” ± 2’ 26” SD (standard deviation)).

Fish in this experiment were maintained and manipulated in accordance with New Zealand National Animal Ethics Advisory Committee guidelines. Ethics applications were approved by the Animal Ethics Committee of Toi Ohomai Institute of Technology and independently reviewed by the University of Otago Animal Ethics Committee.

### Gonadal tissue processing for histology

Histological analysis of gonadal tissues was used to confirm the sex of each individual and identify cellular morphological changes between treatments. Gonads fixed in 4% PFA were processed for routine embedding in paraffin (Histology, Microbiology & Serology Lead, New Zealand Veterinary Pathology, Hamilton Laboratory). Sections were cut at 3 μm and stained with Mayer’s haematoxylin and eosin. Gonadal sections were examined under light microscope and subsequently classified into a series of stages (i.e., female (breeding or non-breeding), early transitioning, mid-transitioning, late transitioning, IP male, and TP male) based on key histological events observed during sex change in socially controlled spotty wrasses [48,49].

### Steroid measurements

Blood was centrifuged at 13,500 rpm for 3 minutes and the plasma supernatant was pipetted off and stored at -20°C until steroid analysis. Measurement of blood plasma concentrations of E2 and 11KT were conducted by radioimmunoassay (RIA) after routine steroid extraction as described in Kagawa et al. [56,57] and Young et al. [58]. Assays were validated for spotty wrasse blood plasma, serially diluted plasma behaving in a manner similar to the standard curve (parallel displacement). Tritiated 11KT was prepared using the methodology described in Lokman et al. [59], whereas label for the E2 assay was purchased from Perkin Elmer. Antiserum against E2 was acquired from MyBioSource and antiserum against 11KT was generously provided by Emeritus Professor Yoshitaka Nagahama, formerly National Institute for Basic Biology, Okazaki, Japan. Following incubation and separation of antibody-bound and -unbound steroid using charcoal-dextran solution (0.5% dextran/charcoal), samples were centrifuged (15 min, 2000g), the supernatant was decanted, and radioactivity was measured using a MicroBeta^®^ Trilux scintillation counter (Wallac 1450, Perkin Elmer). Samples from each experiment were run in separate assays with a minimum detectable level of 140 pg/tube (0.28 ng/mL) for E2 and 15 pg/tube (0.03 ng/mL) for 11KT.

### RNA extraction from gonadal and head kidney tissues

Gonadal and head kidney samples were homogenised using a power homogeniser before RNA extraction. Gonadal RNA was extracted with TRIzol^TM^ (Thermo Fisher Scientific) using chloroform as the phase separation reagent, before DNase-treatment (TURBO DNA-free Kit, Thermo Fisher Scientific) and total RNA clean-up (RNA Clean & Concentrator, Zymo Research). Head kidney RNA was extracted with Direct-zol RNA MiniPrep Plus (Zymo Research) without phase separation (on column DNase treatment). RNA concentration was quantified using a Qubit 2.0 Fluorometer (Life Technologies) and RNA integrity was assessed on a Fragment Analyzer (Advanced Analytical Technologies Inc.).

### Gonadal and head kidney gene expression analysis

For the gonads, a nanoString nCounter^TM^ CodeSet probe array of 19 candidate genes was designed for spotty wrasse as described in Goikoetxea et al. [49] and was used to examine gene expression (S3 Table in S1 File). NanoString gene expression quantification nCounter^TM^ CodeSet was performed at the Otago Genomics Facility, University of Otago, New Zealand. Data were generated using gonadal RNA (100 ng) from 23 control IP fish (day 0, n = 5; day 1, n = 3; day 9, n = 3; day 26, n = 3; day 47, n = 3; day 61, n = 3; day 71, n = 3), 18 cortisol-implanted IP fish (day 1, n = 3; day 9, n = 3; day 26, n = 2; day 36, n = 1; day 47, n = 3; day 61, n = 3; day 71, n = 3), and six control TP males (day 71), samples being chosen based on RNA integrity. Not all gonadal samples collected were included in the NanoString nCounter^TM^ CodeSet gene expression quantification due to funding constraints. Expression data from the full panel of 16 genes were analysed using Principal Component Analysis (PCA) (scaled, R (v. 1.1.453) [60]) to visualise variation in the overall gonadal mRNA expression in relation to cortisol administration. Here, for simplicity, we only present gene expression profiles for five target genes in the gonads relating to the endocrine function of the HPG and HPI axes; among these are two key sex-related genes (*cyp19a1a* and *amh*) and three genes involved in the fish stress response (*cyp11c1*, *nr3c1*, and *nr3c2*) (Table 1). See Supplemental Materials and Methods for details on the complete gene expression results (S6 and S7 Figs in S1 File).

**Table 1:** Genes analysed in spotty wrasse gonad using the nanoString nCounter^TM^ CodeSet technology.

GENE SYMBOL	GENE DESCRIPTION	CONTIG ID	REFERENCE TRANSCRIPT ID
Housekeeping genes			
*actb1*	β-actin, cytoplasmic 1	c58053_g1_i1	NM_131031.1
*eef1a1a*	eukaryotic translation elongation factor 1 alpha 1a	c58053_g1_i1	NM_200009.2
*g6pd*	glucose-6-phosphate dehydrogenase	c39960_g1_i1	ENSDART00000104834.6
Steroidogenesis and hormone receptors			
*cyp19a1a*	aromatase a (gonad isoform)	c52027_g1_i1	NM_131154.3
*cyp11c1/b2*	steroid 11β-hydroxylase	c62027_g1_i1	NM_001080204.1
*nr3c1*	glucocorticoid receptor	c36910_g2_i1	NM_001020711.3
*nr3c2*	mineralocorticoid receptor	c49976_g1_i2	NM_001100403.1
Key sex-related transcription factors			
*Amh*	anti-Müllerian hormone	c51546_g1_i1	NM_001007779.1

For the head kidney, a nanoString nCounter^TM^ PlexSet probe array of 12 candidate genes was designed for spotty wrasse as described in Goikoetxea et al. [49] (Table 2). These genes were chosen based on their steroidogenic function (*cyp19a1a*, *cyp11c1*, *hsd11b2*, *cyp17a1*, *star*, *mc2r*) or on their involvement in the retinoid acid pathway (*cyp26b1*) or epigenetic regulation (*dnmt1* and *dnmt3aa*), respectively, both hypothesised to play a crucial part in hermaphroditic fishes’ gonadal sex change regulation [31,61]. Isolated spotty wrasse sequences were submitted to nanoString Technologies for probe design, PlexSet oligo probes were synthesised by Integrated DNA Technologies (IDT) and nanoString nCounter^TM^ PlexSet gene expression quantification was delivered by the Otago Genomics Facility, University of Otago. Gene expression was examined using head kidney RNA (35 ng) from 26 control IP fish (day 0, n = 5; day 1, n = 3; day 9, n = 3; day 26, n = 3; day 36, n = 3; day 47, n = 3; day 61, n = 3; day 71, n = 3), 20 cortisol-implanted IP fish (day 1, n = 3; day 9, n = 3; day 26, n = 2; day 36, n = 3; day 47, n = 3; day 61, n = 3; day 71, n = 3), and 6 control TP males (day 71), samples being chosen based on RNA integrity. Not all head kidney samples collected were included due to funding constraints during the NanoString nCounter^TM^ PlexSet gene expression quantification. Here, we only present the gene expression profile for *hsd11b2* target gene in the head kidney, see Supplemental Materials and Methods for details on the complete gene expression results.

**Table 2:** Genes analysed in spotty wrasse head kidney using the nanoString nCounter^TM^ PlexSet technology.

GENE SYMBOL	GENE DESCRIPTION	CONTIG ID	REFERENCE TRANSCRIPT ID
Housekeeping genes			
*g6pd*	glucose-6-phosphate dehydrogenase	c39960_g1_i1	ENSDART00000104834.6
*actb1*	β-actin, cytoplasmic 1	c58053_g1_i1	NM_131031.1
*l36*	60S ribosomal protein L36	c40974_g1_i1	NM_001099257.1
Steroidogenesis and hormone receptors			
*cyp19a1a*	aromatase a (gonad isoform)	c52027_g1_i1	NM_131154.3
*cyp11c1/b2*	steroid 11β-hydroxylase	c62027_g1_i1	NM_001080204.1
*hsd11b2*	11β-hydroxysteroid dehydrogenase type 2	c67035_g1_i1	NM_212720.2
*cyp17a1*	steroid 17α-hydroxylase 1	c62160_g1_i1	NM_212806.3
*Star*	steroidogenic acute regulatory protein	c49428_g1_i1	NM_131663.1
*mc2r*	melanocortin 2 receptor	c61860_g3_i1	NM_180971.1
Retinoid Acid (RA) pathway			
*cyp26b1*	cytochrome P450, family 26, subfamily b, polypep. 1	c61802_g1_i1	NM_212666.1
Epigenetic regulatory factors			
*dnmt1*	DNA methyltransferase 1	c43163_g1_i1	NM_131189.2
*dnmt3aa*	DNA methyltransferase 3aa	c59097_g4_i2	NM_001018134.1

The geometric mean of gene pair *actb1*|*g6pd* was selected as the reference for data normalisation for both gonadal and head kidney gene expression results (see Supplemental Materials and Methods for details on the determination of the housekeeping genes’ stability). Normalisation calculations were performed automatically in the nanoString nSolver Analysis software (version 4.0), and normalised data were log base-2 transformed prior to analysis. The nanoString Expression Data Analysis Guidelines (MAN-C0011-04) were followed to determine the expression threshold as described in Goikoetxea et al. [49].

### Statistical analyses

Due to non-normality of the RIA and the normalised nanoString data, the non-parametric Kruskal–Wallis test [62] was used to compare plasma steroid concentrations between treatments, for E2 or 11KT separately, and also to determine whether treatment had a significant effect on each target gene expression level. If treatment was found to have a significant effect, *post hoc* comparisons using Dunn’s tests [63] with Benjamini–Hochberg correction for multiple comparisons [64] were performed between stages to determine where the significance lay, carried out in R (v. 1.1.453) [60].

## Results

### Histological analysis of gonads showed no evidence of cortisol-induced sex change

**Fig 2:**
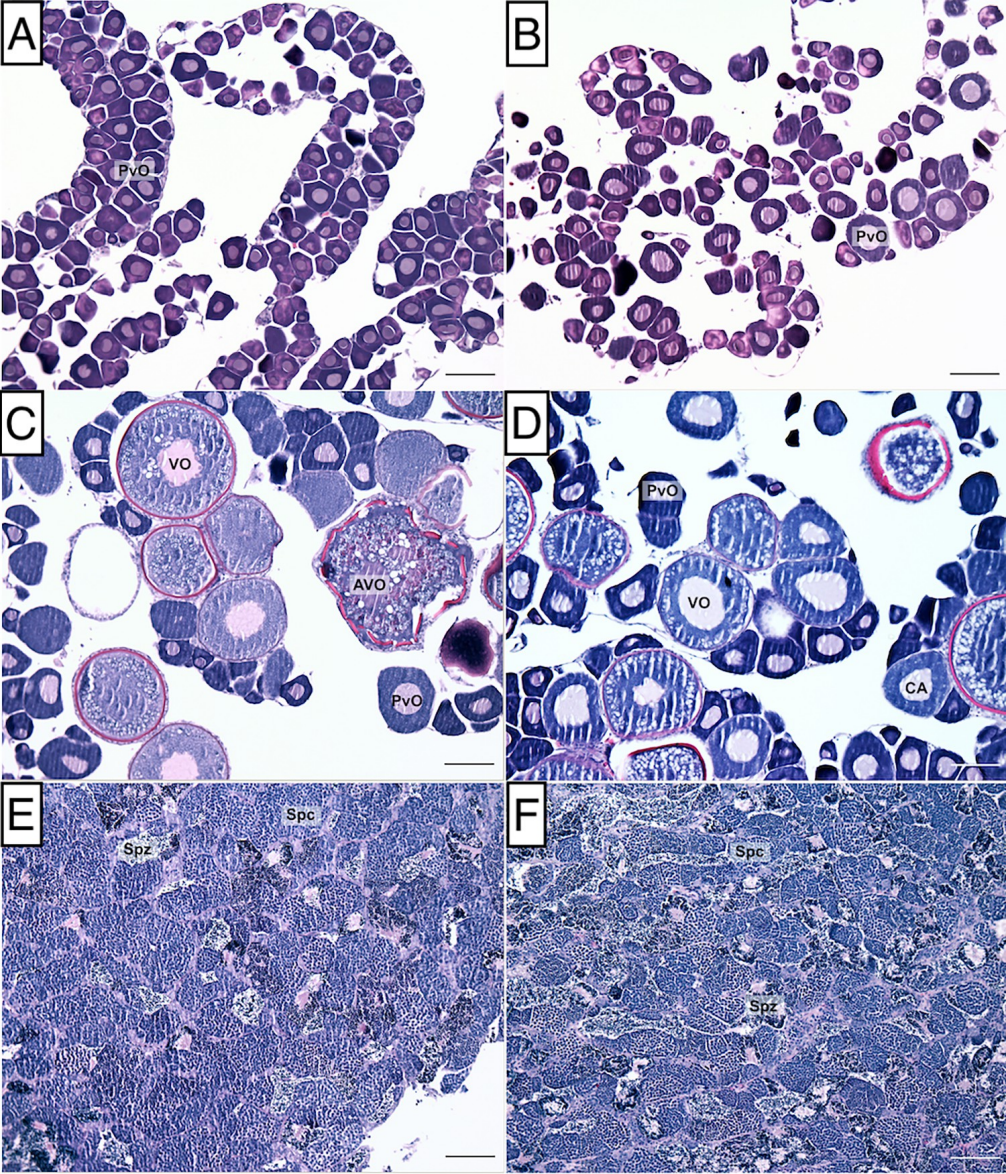
Histological stages of gonadal development observed in spotty wrasses. A) and B) control and cortisol-implanted non-breeding females, respectively, normal ovary filled with previtellogenic oocytes; C) and D) control and cortisol-implanted breeding females, respectively, ovary filled with previtellogenic and vitellogenic oocytes; E) initial phase male, testes containing spermatozoa and cysts of spermatocytes; F) terminal phase male, mature testes showing spermatozoa and cysts of spermatocytes. Abbreviations: atretic oocyte (AVO), previtellogenic oocyte (PVO), spermatocyte (SPC), spermatozoa (SPZ), vitellogenic oocyte (VO). Scale bar: 100 μm.

Histological analysis of the gonads revealed that cortisol treatment did not induce sex change in captive spotty wrasses under socially inhibitory conditions. Out of the 21 cortisol-implanted IP fish, 13 showed regressed ovaries and were classified as non-breeding females (typical of the time of year), while 7 showed signs of maturation consistent with onset of the breeding season and were therefore considered breeding females (Fig 2).

There was also no evidence of sex change within the control tanks (sham implantation, TP male present), except for a single mid-transitioning fish sampled on day 0. Given the larger size of this individual (230 mm) and the progression of sex change, it had likely begun to transition before capture. Otherwise, control fish were classified as non-breeding females with regressed ovaries (n = 18) or breeding females (n = 5), a proportion not statistically significantly different to that of the cortisol-implanted group (*X*^*2*^ (1) = 2.0, p = 0.16).

A single IP individual (cortisol-implanted group; 164 mm TL) was identified as an IP male (2.1% frequency). This is consistent with estimated frequencies of IP males in nature (4.1–5.7%) [46]. Therefore, the IP male found was likely already a male prior to the beginning of the experiment and did not result from premature sex change of an IP female (Fig 2E).

### Plasma concentrations of sex hormones were unaffected by cortisol administration

Of the total number of fish sampled throughout the experiment (n = 54), enough blood plasma (i.e., at least 100 μL) for the E2 steroid analysis was recovered from 47 individuals (n = 4 control fish from day 0, n = 18 control IP fish, 18 cortisol-implanted IP fish, n = 1 IP male, and n = 6 TP males). In the case of the 11KT steroid analysis, enough blood plasma (i.e., at least 100 μL) was available from 41 individuals (n = 3 control fish from day 0, n = 16 control IP fish, 17 cortisol-implanted IP fish, and n = 5 TP males).

For statistical purposes, the single MT found in the control group from day 0 and the single IP male identified in the cortisol-implanted group through the histological analysis of the gonads were excluded from the hormonal profile analysis and subsequent gene expression analysis. Additionally, due to the low number of individuals in the control group from day 0, both control females sampled on day 0 of the experiment, and control females maintained throughout the experiment were grouped altogether as CF (control females) for the purpose of the steroid and gene expression analyses.

**Fig 3:**
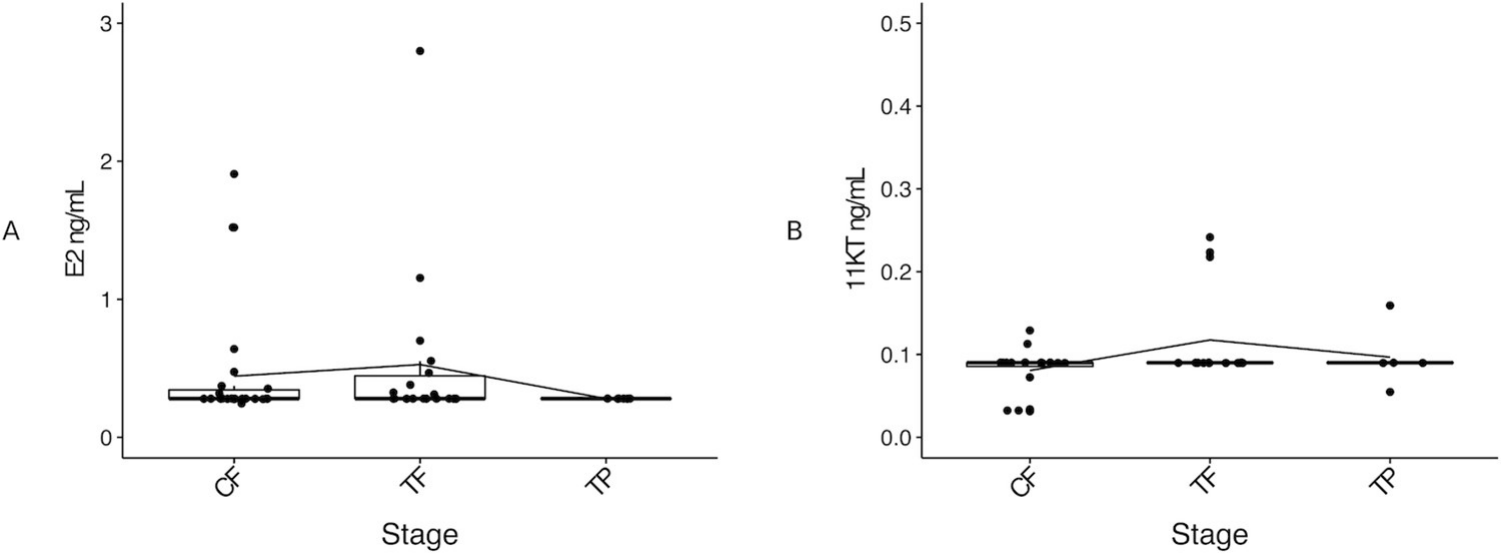
Comparison of plasma E2 (A) and 11KT (B) concentrations between the control females, cortisol-implanted females and TP males. In the boxplots, each point represents an individual fish, the middle bold line represents the median, the edges of the box represent the upper and lower quartiles, and vertical lines represent the minimum and maximum values. Each line represents the variation in mean E2 or 11KT concentration across groups. Sample sizes: E2, CF^☨^ n = 22, TF n = 18, TP n = 6; 11KT, CF n = 20, TF n = 15, TP n = 5. Abbreviations: 11-ketestosterone (11KT), control female (CF), 17β-oestradiol (E2), treatment female (TF), control terminal phase male (TP).

Plasma E2 concentration was not significantly different between cortisol-implanted (treatment females or TF) and CF, or TP males (*X*^*2*^ (2) = 3.44, p = 0.18), with low E2 concentration observed across stages (CF, 0.44 ± 0.42 SD ng/mL; TF, 0.53 ± 0.61 SD ng/mL; TP males, 0.28 ± 0.00 SD ng/mL) (Fig 3A).

Similarly, no significant differences were found among groups for plasma 11KT concentration (*X*^*2*^ (2) = 4.07, p = 0.13; CF, 0.08 ± 0.03 SD ng/mL; TF, 0.11 ± 0.06 SD ng/mL; TP males, 0.10 ± 0.04 SD ng/mL) (Fig 3B).

### A potential impact of cortisol on gene expression in spotty wrasse gonads

**Fig 4:**
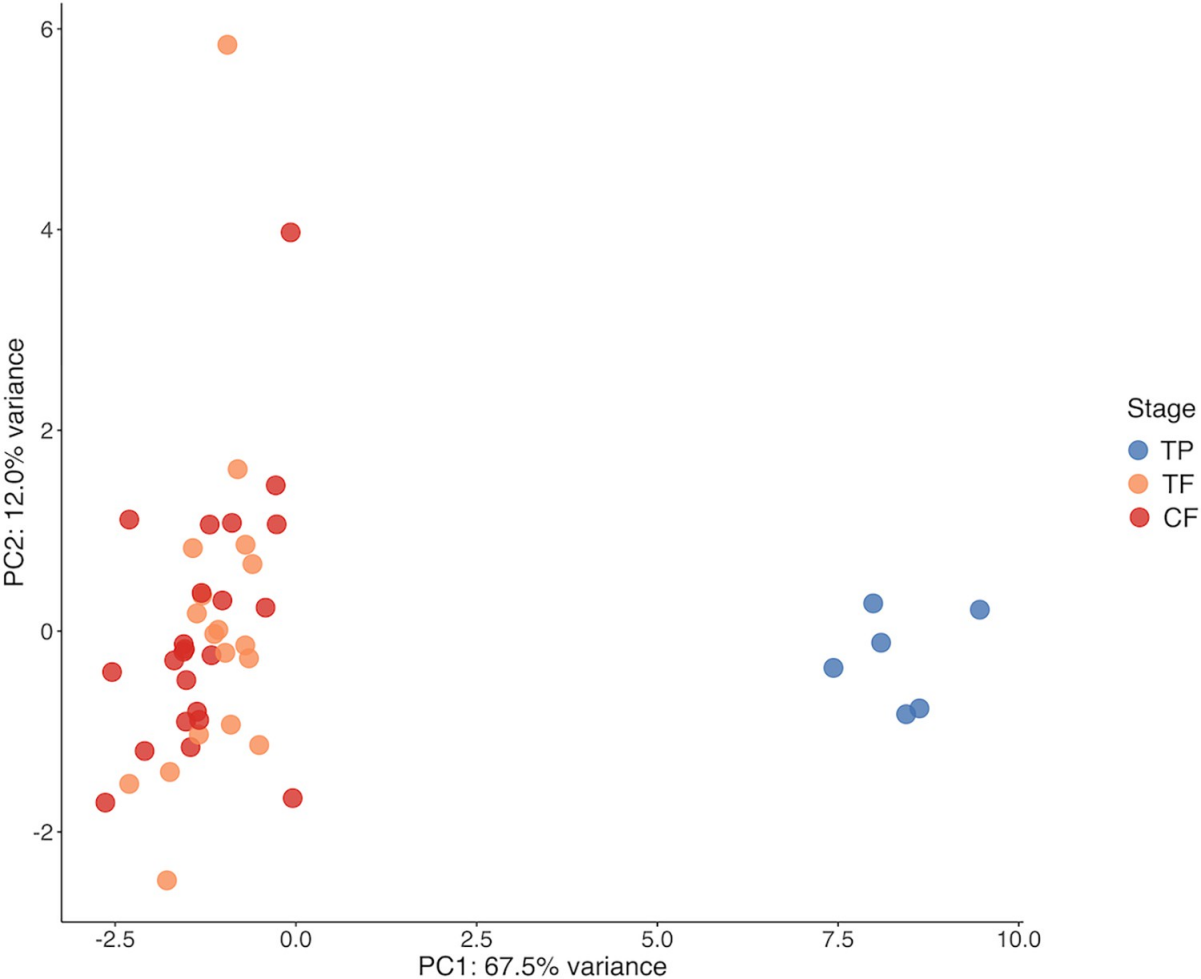
PCA (16 genes) of gonad samples. PC1 (67.5% variance) extremes represent control and cortisol-implanted females (left) and control TP males (right). Sample sizes: CF n = 23, TF n = 18, TP n = 6. Abbreviations: control female (CF), treatment female (TF), control terminal phase male (TP).

The lack of transitional spotty wrasses obtained through the implantation of cortisol pellets prevented comparisons of expression patterns across sex change. Therefore, gene expression was compared between CF, TF and control TP males. For statistical purposes, the single MT and IP male individuals identified in the control and cortisol-implanted groups, respectively, were excluded from expression analyses. PCA clustering of samples based on the collective gene set revealed that gonadal samples were strongly clustered by sexual stage (PC1, 67.5% variation explained), with both CF and TF clearly grouped together, and separate from their male counterparts (Fig 4).

**Fig 5:**
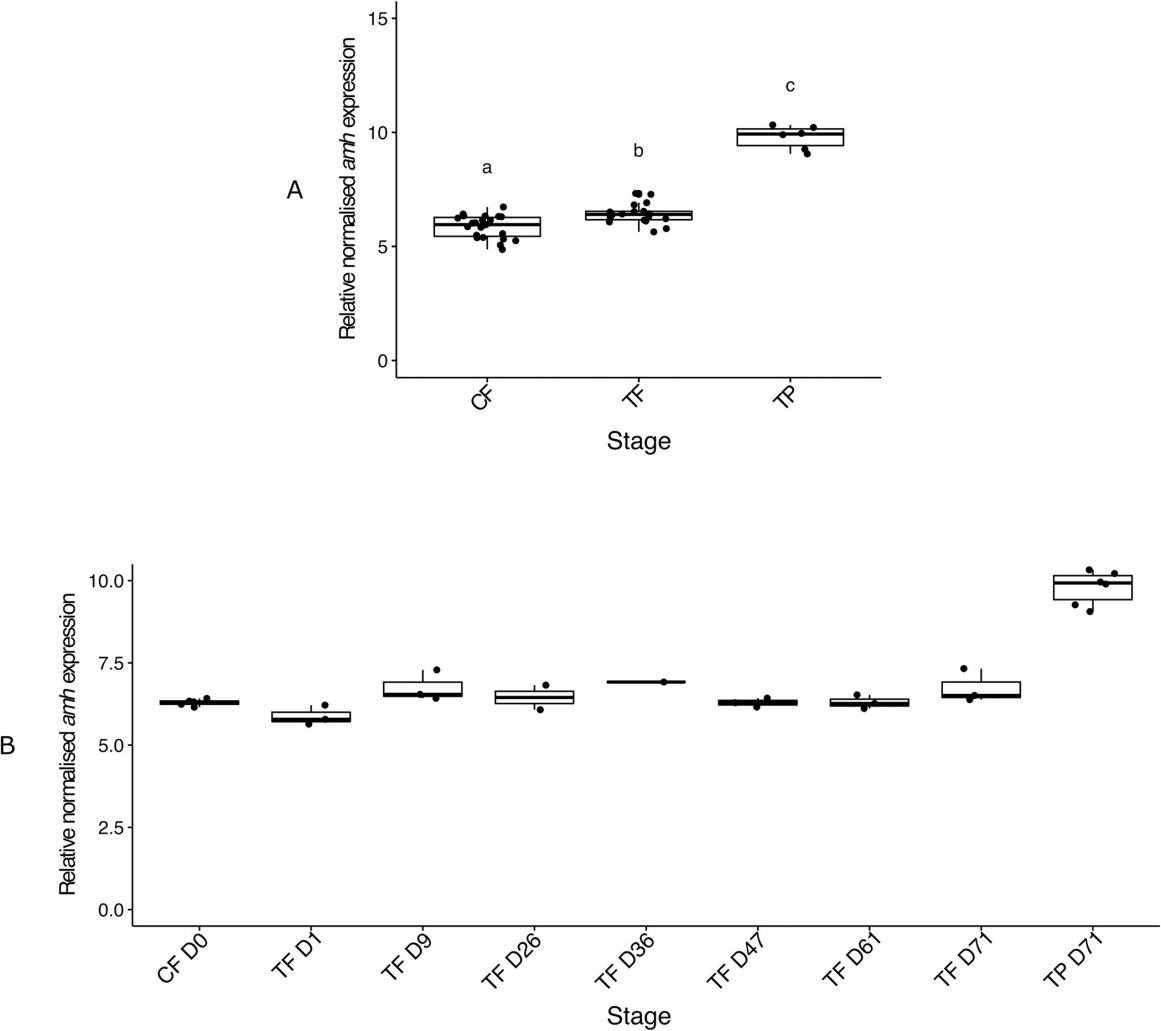
Relative gonadal expression of *amh* mRNA compared collectively among control females, cortisol-implanted females and TP males (A) and serially among control females from day 0, cortisol-implanted females sampled on 7 time points (days 1, 9, 26, 36, 47, 61 and 71) and TP males sampled at the end of the experiment (B). In the boxplots, each point represents an individual fish, the middle bold line represents the median, the edges of the box represent the upper and lower quartiles, and vertical lines represent the minimum and maximum values. Letters denote significant differences between groups. Sample sizes: CF n = 23, TF n = 18, TP n = 6, CF D0 n = 5, TF D1 n = 3, TF D9 n = 3, TF D26 n = 2, TF D36 n = 1, TF D47 n = 3, TF D61 n = 3, TF D71 n = 3, TP D71 n = 6. Abbreviations: control female (CF), day (D), treatment female (TF), control terminal phase male (TP).

There was an effect of sex stage on *amh* (anti-Müllerian hormone) expression (*X*^*2*^ (2) = 23.74, p < 0.001) (Fig 5A), with *amh* expression being TP male-biased as expected (TP males median 1.67-fold higher than CF and 1.52-fold higher than TF). *Amh* expression was also significantly higher in the TF than CF (TF median 1.10-fold higher than CF) suggesting cortisol treatment induced *amh* upregulation in TF. When including each sampling day, a subtle increase in *amh* expression was observed on day 9 post implantation. However, our analyses were constrained by low sample sizes and low statistical power and no statistically significant differences between conditions were observed (Fig 5B).

**Fig 6:**
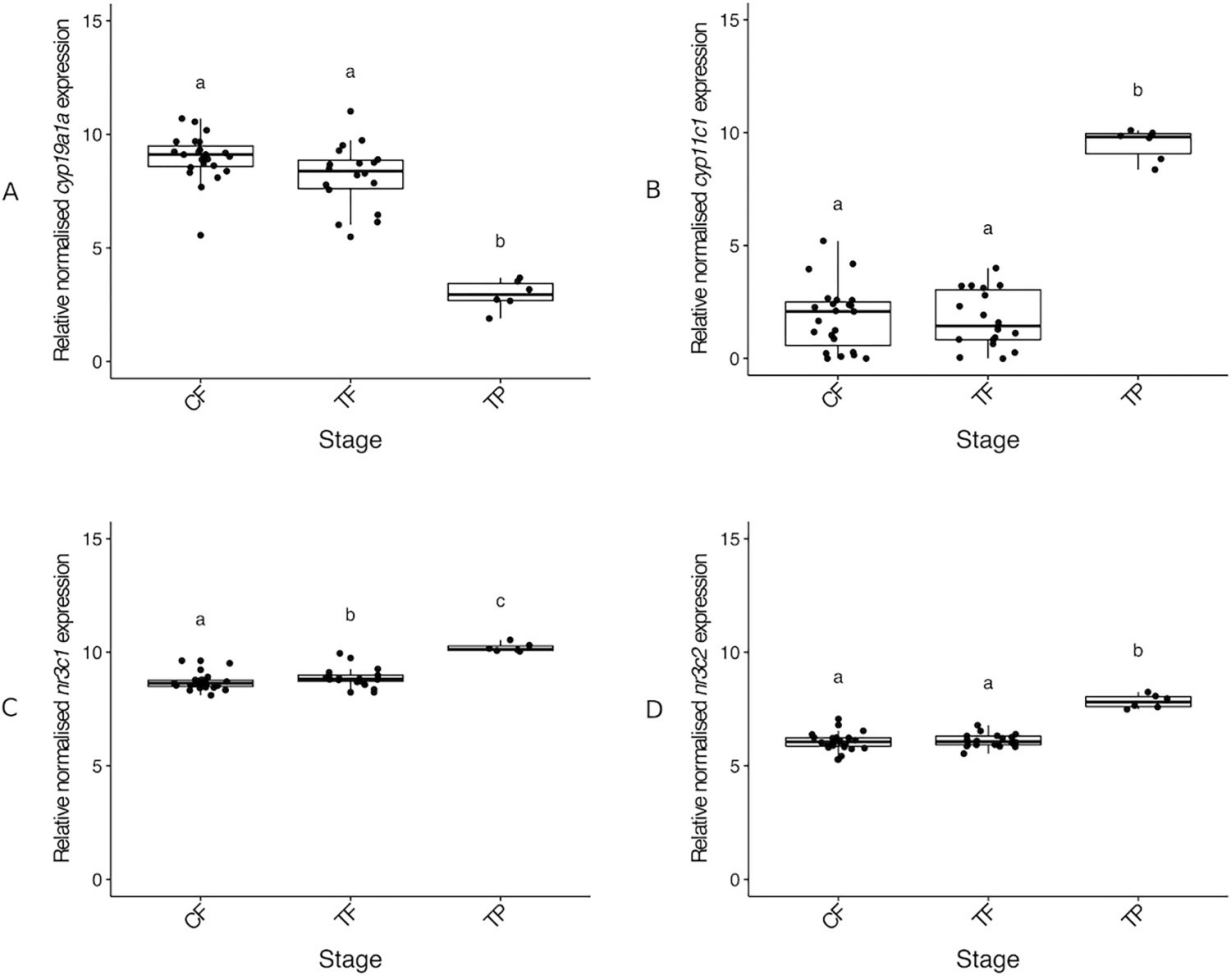
Relative gonadal expression of *cyp19a1a* (A), *cyp11c1* (B), *nr3c1* (C) and *nr3c2* (D) mRNA. Expression levels are compared among control females, cortisol-implanted females and TP males. In the boxplots, each point represents an individual fish, the middle bold line represents the median, the edges of the box represent the upper and lower quartiles, and vertical lines represent the minimum and maximum values. Letters denote significant differences between groups. Sample sizes: CF n = 23, TF n = 18, TP n = 6. Abbreviations: control female (CF), treatment female (TF), control terminal phase male (TP).

Expectedly, *cyp19a1a* expression was significantly higher in the CF and TF compared to TP males (median 3.05-fold and 2.77-fold higher than TP, respectively) (*X*^*2*^ (2) = 18.83, p < 0.001) (Fig 6A). Expression of *cyp11c1* (11β-hydroxylase) also showed the expected sex-biased expression towards TP males, with a significantly higher expression in TP males compared to the control (median 5.26-fold higher than CF) and cortisol-implanted females (median 5.44-fold higher than TF) (*X*^*2*^ (2) = 15.38, p < 0.001) (Fig 6B). Significant differences between TP males and CF were also observed in the expression of *nr3c1* (encoding glucocorticoid receptor) and *nr3c2* (encoding mineralocorticoid receptor) (*nr3c2*, *X*^*2*^ (2) = 19.93, p < 0.001; *nr3c2*, *X*^*2*^ (2) = 15.56, p < 0.001) (Fig 6C and 6D).

### Weak evidence for an effect of cortisol on gene expression in spotty wrasse head kidneys

**Fig 7:**
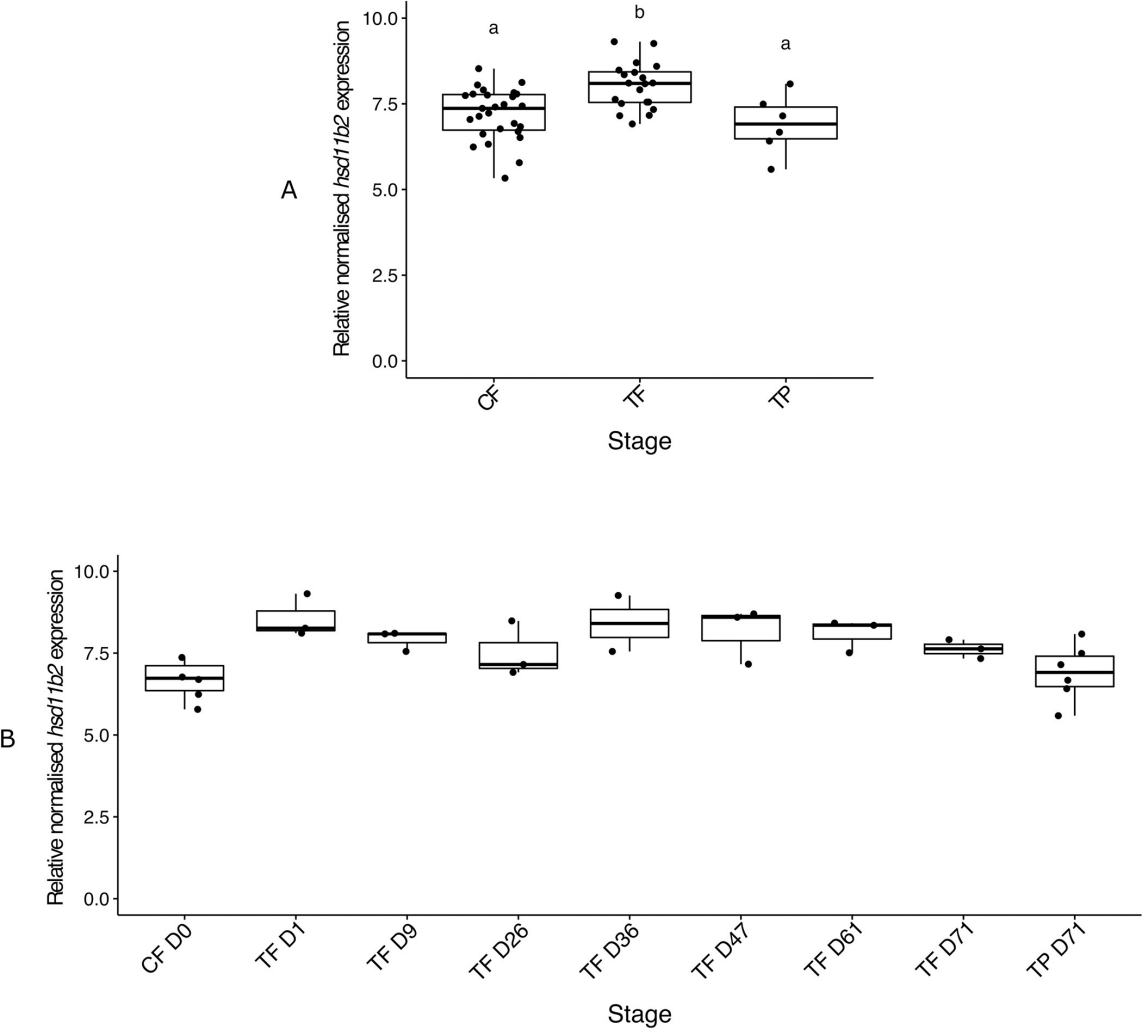
Relative head kidney expression of *hsd11b2* mRNA compared collectively among control females, cortisol-implanted females and TP males (A) and serially among control females from day 0, cortisol-implanted females sampled on 7 time points (days 1, 9, 26, 36, 47, 61 and 71) and TP males sampled at the end of the experiment (B). In the boxplots, each point represents an individual fish, the middle bold line represents the median, the edges of the box represent the upper and lower quartiles, and vertical lines represent the minimum and maximum values. Letters denote significant differences between groups. Sample sizes: CF n = 27, TF n = 20, TP n = 6, CF D0 n = 5, TF D1 n = 3, TF D9 n = 3, TF D26 n = 3, TF D36 n = 2, TF D47 n = 3, TF D61 n = 3, TF D71 n = 3, TP D71 n = 6. Abbreviations: control female (CF), treatment female (TF), control terminal phase male (TP).

A significant difference in *hsd11b2* (11β-hydroxysteroid dehydrogenase type 2) head kidney expression was found between control females and TP males compared to cortisol-implanted females, but not between CF and TP males (TF median 1.11-fold higher than CF and 1.16-fold higher than TP males) (*X*^*2*^ (2) = 14.16, p < 0.001) (Fig 7A). When including all sampling days for cortisol-implanted females, there appeared to be a trend of increased *hsd11b2* expression at day 1 (TF day 1 median 3.7-fold higher than CF from day 0), but low sample sizes per condition prevented a reliable statistical analysis from being performed (Fig 7B).

## Discussion

An increased understanding of the process of sex change in hermaphroditic fish, and particularly the role of stress in this process, will help us gain further insight into sex determination and differentiation processes in other vertebrates, including humans. A better understanding of these processes may provide novel approaches to the control of sexual differentiation, and provide opportunities in agriculture and aquaculture. In the latter, the prevention of precocious maturation, production of monosex populations, and greater control over brood stocks may generate significant economic benefits [65]. In this study, we simulated the potential role of stress in the induction of female-to-male sex change by administering cortisol to spotty wrasse under conditions socially non-permissive to sex change. Gonadal histological analysis, measurement of plasma sex hormones and evaluation of gene expression in the gonad and head kidney were used to identify any induction of sex change.

### Cortisol administration did not induce sex change in non-permissive conditions

We found no histological evidence for sex change in cortisol-implanted female spotty wrasse. Had sex change occurred in the cortisol-implanted females, we would have expected the presence of atretic oocytes and nests of gonial cells in early sex changers, and evidence of spermatogenic germ cell cysts in mid- and late stages of sex change [48,49]. Protogynous sex change has been promoted via cortisol administration, in the three-spot wrasse [15] and the sandperch [21], with evidence of sex change observed at the histological level in both studies. Further, an increase in serum cortisol levels was reported during the early stages of natural female-to-male sex change in the bidirectional bluebanded goby (*Lythrypnus dalli*) [14]. In spotty wrasse, sex change under laboratory conditions has previously been achieved via a single fadrozole (an aromatase inhibitor) implantation [49] or social manipulation (i.e., removal of the dominant male from the social group) [48,49]. In both cases, sex change was promoted with a success rate of at least 80% and signs of full sex change were evident in under 70 days [48,49]. In this experiment, unseasonally poor weather impeded fish collection and delayed the start of the experiment. As a result, we started our experiments in June, later than when sex change in the wild is known to proceed (November to May) [46], which we have subsequently discovered is outside the optimal window for sex change in our captive system [49]. This was reflected in the histological analysis of the gonads which showed that the ovaries of 21.7% of CF and 35% of TF were approaching breeding condition, with vitellogenic oocytes present (Fig 2C and 2D). Thus, cortisol may not have triggered sex change in spotty wrasse in this study due most likely to the timing of cortisol administration (i.e., outside of the optimal window for natural sex change).

In previous studies, cortisol was administered via food (200 μg/g or 1,000 μg/g of pellet food for 6 weeks for the three-spot wrasse [15]; 300 mg/kg feed for 84 days for the black sea bass [22]) or via implantation (50 μg/g body weight during 21 days for the sandperch [21]). Cortisol administration via food makes determining the dosage of cortisol received by each individual difficult. Here, we administered cortisol via pellet implantation, thus each fish received a known amount of cortisol (5 mg/implant, i.e., an average of 106.2 ± 18.8 μg/g body weight). It is challenging to draw parallels between those cortisol concentrations mixed in the food and our implanted dose, however our employed dose was double that used by Frisch et al. in the sandperch [21]. Nevertheless, the duration of cortisol treatment was shorter compared to previous studies; here, the majority of cortisol was released from the pellets in the first 8–10 days (see Supplemental Materials and Methods), whereas previous experiments using cortisol in feed allowed continual cortisol treatment for 21 days [21], 6 weeks [15], and 84 days [22]. The involvement of cortisol has also been explored during temperature-induced masculinization of several gonochoristic species. Successful promotion of sex change with cortisol treatment was accomplished in the pejerrey (0.4 mg cortisol/g diet up to 7 weeks) [20], medaka (5x10^-6^ M cortisol from 0 to 5 days post hatching (dph)) [16], Japanese flounder (10 or 100 μg/g diet of cortisol from 30 to 100 dph) [18], or Southern flounder (0, 100, or 300 mg cortisol/kg of gelatin-coated feed until 42 days post stocking) [19]. However, in these works, cortisol involvement in gonochoristic sex differentiation was analysed in the larvae [16,18,20] or juvenile [19] phases, and sensitivity to cortisol administration in these systems may differ greatly from that of hermaphroditic teleosts, which change sex naturally and during adulthood. Future experiments administering cortisol outside of the breeding season and with a more prolonged release of cortisol will be useful to determine whether cortisol administration is sufficient to override social inhibition to induce sex change.

Unexpectedly, we did not observe lower values of E2 and higher values of 11KT in control TP spotty wrasse males compared to females. A lower than anticipated steroid extraction recovery rate of E2 and 11KT from spotty wrasse plasma (E2, 65%; 11KT, 45%) for reasons that are not clear may have prevented the measurement of true plasma sex steroid concentrations. As the experiment overlapped with the breeding season (July to late October), when higher E2 and 11KT concentrations may be expected [49], this further suggests that the extraction procedure, rather than naturally low sex hormone levels, may have affected the outcome of the assay. This possibility is further reinforced by the higher levels of E2 observed in the TF classified as breeding females in comparison to those considered non-breeding females (median 3.22-fold higher) found in the present experiment.

The average time from capture to blood collection in the fish (i.e., 6’ 0” ± 2’ 26” SD) indicated that plasma cortisol concentration at the time of blood collection would have been largely affected by handling [53–55]. Moreover, we cannot dismiss the possibility that confinement itself (i.e., a three-week acclimation) may have acted as a chronic stressor, affecting the potential measurement of cortisol levels [66]. For these reasons, we did not measure plasma cortisol levels. In future experiments, handling-induced increases in cortisol levels may be avoided by using a closed seawater system, adding the anaesthetic agent directly in the water, and applying a whole-tank sampling regime to minimise stress caused by handling.

### Upregulation of *amh* and *nr3c1* suggest cortisol directly impacts gonadal gene expression

Our gene expression analysis in the gonads showed sex-specific expression of all genes. *Cyp19a1a*, *cyp11c1* and *nr3c2* showed no change in expression between control and cortisol-implanted females, whereas *amh* and *nr3c1* showed a significant increase in cortisol-implanted, compared to control, females, indicating a potential limited masculinising effect of cortisol at the molecular level.

Evidence that cortisol treatment may have had a masculinising effect on gene expression was seen for the *amh* gene. Cortisol is hypothesised to induce maleness by upregulating *amh* expression, which can then induce germ cell apoptosis to promote masculinisation [6,18,20,29,30,34, reviewed by 67]. Moreover, our previous work has demonstrated that *amh* is upregulated across sex change in spotty wrasse and this is evident at the early stages of sexual transition [49]. These data provided a foundation establishing *amh* as a potential proximate activator of masculinisation in this species [49]. In the present work, in TF at day 9 *amh* expression reached levels intermediate to those of CF and TP males, and remained at this level in TF across all remaining sampling days. As we did not observe a decrease in *amh* expression levels following its peak on day 9 (when most of the hormone was likely released from the implants; see S1 Fig in S1 File), this suggests that cortisol may have succeeded in pushing *amh* expression towards maleness. Despite the significant increase in *amh* expression observed, it should be noted that such a rise was very small. Therefore, based on the present data and low sample sizes per sampling day we can only hypothesize about the biological relevance of the reported changes. Thus, our data provide some evidence that cortisol may be involved in spotty wrasse sex change and is conceivably part of its initiation mechanism. However, our results showed that the change in *amh* expression alone was not enough to activate the genetic cascade orchestrating the ovary-to-testis transformation.

Both *nr3c1* and *nr3c2*, which code for the glucocorticoid and mineralocorticoid receptor, respectively, showed a male-biased expression, in line with observed patterns during sex change via social induction in the same species [49]. *Nr3c1*, but not *nr3c2*, showed an increase in expression in cortisol-implanted, compared to control, females. The upregulation of *nr3c1* in cortisol-implanted spotty wrasse suggests, along with *amh* upregulation, a slight masculinising effect of cortisol. Increased expression of *nr3c1* in cortisol-implanted spotty wrasses was unexpected, because multiple studies in teleost fishes have reported a decline in Nr3c1 as a response to high plasma cortisol concentrations [68–71]. However, cortisol-induced upregulation of *nr3c1* mRNA abundance also occurred in the rainbow trout (*Oncorhynchus mykiss*) [72]. Thus, the effect of cortisol on *nr3c1* may vary in a species-specific fashion.

Conversely, *cyp19a1a* showed an expected significantly female-biased expression in the gonad. However, we found no difference in *cyp19a1a* expression between cortisol-implanted and control females, which correlates with the lack of differences in blood plasma E2 concentrations between CF and TF individuals. A lack of change in *cyp19a1a* expression after cortisol administration further suggests cortisol does not induce sex change in spotty wrasse or was not enough to promote sex change alone.

For *cyp11c1* (11β-hydroxylase), a significantly male-biased expression in the gonad was observed. However, no changes in expression were found between control and treatment females, in agreement with the absence of differences in 11KTconcentrations observed between these two groups. Our results agree with previous observations in the same species of higher *cyp11c1* expression in TP males and transitioning individuals socially induced to change sex compared to control females [49]. The lack of significant differences between CF and TF also supports the potential inability of cortisol to promote sex change in this experiment implied by the *cyp19a1a* data. The overall pattern of gonadal gene expression we observed suggests that although cortisol treatment may have ‘nudged’ gene expression towards maleness, its effect was insufficient to initiate the full genetic cascade required for gonadal sex change.

### Head kidney *hsd11b2* expression indicates a physiological effect of the cortisol implants

Given that gene activity in the interrenal tissue of sex changing fish is vastly understudied, and considering that the head kidney is the major site of cortisol synthesis in teleosts [73], we also measured gene expression in the head kidney of spotty wrasse. Enzyme Hsd11b2 is involved in the 11-oxyandrogen biosynthetic pathway, and is also responsible for inactivating cortisol, by 11-oxidising it to cortisone [reviewed by 29,74]. However, Hsd11b2 is not considered a limiting factor in 11KT synthesis, and therefore an increase in *hsd11b2* expression may not be associated with cortisol-mediated androgen production. In fact, there is evidence in both mammals and fishes of an ultra-short-loop negative feedback by which cortisol would suppress its own synthesis [75,76], meaning that increased levels of cortisol would lead to its conversion to cortisone, its inactive form [77]. This mechanism is hypothesised to be driven by Hsd11b2, with a widespread tissue distribution, which would be responsible for the peripheral conversion of cortisol to cortisone in order to protect the gonads from the harmful effects of high circulating corticosteroids [77,78]. Our data suggest that cortisol administration experimentally increased the levels of *hsd11b2* in spotty wrasses and possibly promoted the activation of the Hsd11b2-mediated inhibitory loop to counteract the high levels of cortisol. The effect of cortisol on *hsd11b2* was predicted to be stronger during the first 8–10 days of the experiment and then to become progressively downregulated due to the majority of cortisol being released from the pellets in the first 8–10 days (see Supplemental Materials and Methods). However, we saw a peak in *hsd11b2* on day 1 with expression remaining at a similarly high level in cortisol-implanted females for the remainder of the experiment. The function of *hsd11b2* in the head kidney is still not fully understood [77], and its potential role in androgen production by interrenal cells cannot be conclusively rejected. Hence, the cortisol-induced increase in *hsd11b2* may reflect a response to cope with high cortisol levels but may also indicate a weak masculinising effect of cortisol.

## Conclusions

In this study, short exposure (8–10 days) to one-off high levels (5 mg) of cortisol did not induce sex change in captive protogynous New Zealand spotty wrasse females under non-permissive social conditions. However, despite a lack of histological and steroidal evidence for sex change, nanoString-generated molecular data suggests that cortisol treatment may have had a weak masculinising effect on expression of key sex- and stress-related genes. Expression of gonadal *amh*, considered to be cortisol-responsive and an early mediator of sex reversal, was significantly higher in cortisol-implanted females compared to controls. In the head kidney, *hsd11b2* expression was upregulated in response to cortisol administration, again suggesting there was a physiological effect of cortisol implants.

Conceivably, although cortisol alone may be unable to induce sex change in spotty wrasse, it is part of a complex cascade involving multiple factors to ensure sex change proceeds only under optimal circumstances. Alternatively, the duration of the cortisol treatment applied may have been insufficient to promote masculinisation. Furthermore, the seasonality of the experiment (i.e., within the breeding season of spotty wrasse) may have reduced the physiological responsiveness of females to sex change cues and thus the effectiveness of cortisol implants to promote sex change, as this transformation occurs naturally in the post-spawning period. Future manipulative experiments involving a long-term exposure to high cortisol (e.g., using slow-release cortisol pellets, mixing the hormone directly in the diet or through the use of osmotic minipumps) and performed outside the breeding season are necessary to address these issues and further test the role of stress during socially induced sex change in protogynous fishes.

## Supporting information

S1 File Supplemental materials and methods–contains all the supporting tables and figures. (DOCX)
