# Supplementary material for: Effects of cortisol on female-to-male sex change in a wrasse
Source: PLoS One. 2022 Sep 1;17(9):e0273779. doi: 10.1371/journal.pone.0273779 (PMC9436091; doi:10.1371/journal.pone.0273779)
Supplement: S1 File — (DOCX) [file pone.0273779.s001.docx]

# Supplementary Material

# Cortisol pilot study

## Introduction

The release rate of cortisol from implants containing different concentrations was tested both *in vivo* and *in vitro* in order to establish a protocol to evaluate the hypothesis tested in this study. The aim was to quantify the release rate of the cortisol pellets.

## Materials and Methods

### In vivo experiment

To measure the release rate of cortisol pellets *in vivo*, eight initial phase (IP) and four terminal phase (TP) spotty wrasses were captured and subsequently maintained for 29 days during March 2017 as described in this study. Fish were evenly distributed across four 400-litre recirculating seawater systems (2 IP fish per tank) and natural sex change was blocked by placing a TP male in each tank.

Pellets containing 50, 500 and 5000 μg of cortisol (Sigma-Aldrich), respectively, were made in-house as described in this study. The choice of dosages to be tested took into consideration multiple studied involving cortisol administration in different species found in the literature, among which a great variability was observed [1–8]Control pellets (vehicle) contained matrix only. On day 0, IP fish were colour-tagged for individual identification, given a single intramuscular implant and blood samples were collected as described in this study. During sampling, each pair of IP individuals was captured simultaneously not to confound stress levels and as quickly as possible to minimise stress caused by handling.

Blood samples were collected on days 1, 8, 15 and 29 from each IP fish. On day 29 (end of experiment), all fish were terminally sampled and one gonad from each fish was preserved for histological analysis as described in this study. Water samples were also taken from each tank before implantation (day 0) and on days 1, 2, 6, 8, 13, 15, 22 and 29 throughout the experiment. This was done to ascertain whether cortisol is excreted into the tank water, with the potential for transfer to neighbouring treatment tanks because of the recirculating aquaculture system (RAS). Fish were maintained and manipulated in accordance with New Zealand National Animal Ethics Advisory Committee guidelines (approved by the Animal Ethics Committee of Toi Ohomai Institute of Technology).

Gonadal tissues were fixed and processed for histology as described in this study. Plasma cortisol (CORT) concentrations were measured via radioimmunoassay (RIA) and subsequent statistical analysis of results were performed as described in this study.

### In vitro experiment

Release rate of cortisol implants *in vitro* was also evaluated. Pellets containing 0 (control), 50, 500 and 5000 μg of cortisol, made in-house as described in this study, were each placed on an L-shaped piece of coarse plankton mesh folded into a cone shape and inserted into a 50 mL Falcon^®^ tube containing 10 mL of salmon Ringer’s solution (prepared as described in [9]), which has a similar osmolality to that of spotty wrasses’ plasma [10]. Three tubes of each cortisol concentration were run (n = 12 (3 x 4)). Tubes were agitated on a shaker table for 32 days. Samples (3 x 1-mL aliquots; n total =36 (3 x 12)) from the Ringer’s solution were taken every day, and the remaining Ringer’s (7 mL) discarded and replaced with 10 mL of fresh solution (to mimic the metabolism of the fish).

HPLC (high-performance liquid chromatography) was used to measure both the release rate of the cortisol pellets *in vitro* (days 1, 2, 4, 8, 12, 16, 20, 24, 28, 32) and cortisol concentration in the tank water from the *in vivo* experiment (days 0, 1, 2, 6, 8, 13, 15, 22, 29). Twenty microlitres of sample were injected onto a pre-calibrated Agilent Technologies 1200 Series HPLC equipped with an Agilent Eclipse XDB-C18 column (4.6 mm × 150 mm; 5 µm) and using ultra-violet (240 nm). Triplicates for each sample were analysed (n = 12 (3 x4)). Samples were run at a flow rate of 1 mL/min. Statistical analysis was performed as described in this study.

## Results

Histological analysis of the gonads did not reveal any significant differences in gonadal morphology between cortisol-implanted fish and controls. All six cortisol-implanted fish were females in a non-breeding state (NBF).

Radioimmunoassay CORT measurement demonstrated that plasma cortisol levels in the *in vivo* experiment decreased dramatically after day 8 post-implantation for all pellet concentrations (50, 500 or 5000 μg). The highest cortisol peak for all treatments was detected on day 1: 50 μg, 455.05 ± 424.12 SD ng/mL; 500 μg, 85.46 ± 20.86 SD ng/mL; and 5000 μg, 610.87 ± 409.33 SD ng/mL (Fig S1); with the highest mean cortisol concentration detected in plasma from fish implanted with the highest cortisol concentration (5000 μg pellets) (*X^2^* (4) = 15.90, p < 0.005). Cortisol levels in wild spotties have been reported to fluctuate between 2 – 208 ng/mL [11]. The time from capture to blood extraction from the fish averaged 4’ 21’’ ± 1’ 15’’ SD. Blood collection in under 3 minutes was only achieved in 21% of cases, and plasma cortisol levels during the *in vivo* assay might have been affected by handling [12–14].

Cortisol released into the tank water, as measured by HPLC, was negligible and no significant changes were observed in water samples after implantation of the pellets compared to initial control samples (Fig S2), suggesting neighbouring tanks were not affected by cortisol excreted into the recirculating water system (*X^2^* (8) = 6.76, p = 0.56).

Cortisol release from pellets measured *in vitro* by HPLC correlated well with the *in vivo* results for blood plasma measured by RIA. HPLC showed that in all cases, cortisol levels in the salmon Ringer’s solution decreased dramatically after day 8. Likewise, the highest cortisol peak for all treatments was detected on day 1: 50 μg, 3.80 ± 0.32 SD ng/mL; 500 μg, 15.99 ± 1.76 SD ng/mL; and 5000 μg, 64.88 ± 4.20 SD ng/mL (*X^2^* (9) = 8.71, p = 0.46) (Fig S3). The highest mean cortisol concentration was detected in the salmon Ringer’s containing the highest cortisol concentration (5000 μg) pellets.

Thus, the 5000 μg concentration cortisol pellet was selected for the cortisol administration experiment from the present study.

# Determination of housekeeping genes’ stability (gonad)

The ranking of gonadal candidate reference genes by RefFinder [15], Δ CT and NormFinder [16] was *actb1* > *g6pd* > *eef1a1a*. The BestKeeper [16] ranking was *g6pd* > *actb1* > *eef1a1a*, based on both SD and r. (Tables S1A and S1B). These results suggest that *actb1* and *g6pd* should be used as reference genes for nanoString data normalisation.

We also assessed stability of housekeeping genes by using the non-parametric Kruskal–Wallis test [17] to determine if there was a significant effect of treatment on the candidate reference genes. The gonadal mRNA levels of candidate reference genes *eef1a1a* and *g6pd* were found to be significantly affected by treatment using the non-parametric Kruskal–Wallis test (*eef1a1a*, *X^2^* (2) = 6.53, p < 0.05; *g6pd*, *X^2^* (2) = 9.76, p < 0.01). The geometric mean of gene pair *actb1*|*eef1a1a* was also significantly influenced by treatment (*X^2^* (2) = 7.01, p < 0.03). Candidate reference gene *actb1* mRNA levels (*X^2^* (2) = 5.91, p = 0.05) and the geometric mean of mRNA levels of the following combination of genes were not significantly affected by treatment; *actb1*|*g6pd* (*X^2^* (2) = 0.03, p = 0.99), *eef1a1a*|*g6pd* (*X^2^* (2) = 0.91, p = 0.63), and *actb1*|*eef1a1a*|*g6pd* (*X^2^* (2) = 0.80, p = 0.67) (Fig S4). Consequently, any of the latter candidate housekeeping gene combinations can potentially be used for normalisation of nanoString data. Gene pair *actb1*|*g6pd* was selected to normalise the target gene expression data (Fig S4E).

# Determination of housekeeping genes’ stability (head kidney)

The ranking of head kidney candidate reference genes by RefFinder [15], Δ CT and NormFinder [16] was *g6pd* > *l36* > *actb1.* In contrast, BestKeeper [16] found *l36* to be the most stable housekeeping gene (*l36* > *g6pd* > *actb1*), based on both SD and r. (Tables S2A and S2B). These results suggest that *g6pd* and *l36* should be used as reference genes for nanoString data normalisation.

Again, we also used the non-parametric Kruskal–Wallis test [17] to assess the stability of housekeeping genes. Candidate reference gene *actb1* (*X^2^* (2) = 2.78, p = 0.25), *g6pd* (*X^2^* (2) = 2.71, p = 0.26) and *l36* (*X^2^* (2) = 0.82, p = 0.66) mRNA levels and the geometric mean of mRNA levels of all possible combination of genes were not significantly affected by treatment; *actb1*|*g6pd* (*X^2^* (2) = 2.62, p = 0.27), *actb1*|*l36* (*X^2^* (2) = 1.77, p = 0.41), *g6pd*|*l36* (*X^2^* (2) = 2.20, p = 0.33) and *actb1*|*g6pd*|*l36* (*X^2^* (2) = 2.62, p = 0.27) (Fig S5). Consequently, any of these candidate housekeeping gene combinations can potentially be used for normalisation of nanoString data. However, the expression of *l36* gene was very low across all treatment groups (Fig S5C), and therefore any gene combination including this gene was excluded from consideration as reference. The analysis of candidate reference genes performed by Kruskal–Wallis suggests that gene pair *actb1*|*g6pd* should be the most stable (i.e., highest p-value for a gene combination observed) and show the flattest profile across gene combinations (Fig S5D). For this reason, gene pair *actb1*|*g6pd* was selected to normalise the target gene expression data.

# Head kidney gene expression analysis

## Results

Expression of *star* (steroidogenic acute regulatory protein) gene was not significantly affected by cortisol treatment (*X^2^* (2) = 2.63, p = 0.29) (Fig S8A). Epigenetic regulatory factors *dnmt1* and *dnmt3aa* were also not affected by cortisol treatment (*dnmt1*, *X^2^* (2) = 1.93, p = 0.38; *dnmt3aa*, *X^2^* (2) = 0.10, p = 0.95) (Figs S8B and S8C). Several genes with expression below the detection threshold across head kidney samples were excluded from analysis, which included *cyp19a1a*, *cyp11c1*, *cyp17a1*, *mc2r* and *cyp26b1*.

## Discussion

Cortisol treatment did not affect *star* (steroidogenic acute regulatory protein) expression, which encodes homonymous Star, a transport protein that controls cholesterol (from which cortisol is synthesised) transfer within the mitochondria [18]. The lack of changes in *star* expression indicates that any physiological effect of cortisol was not prolonged or strong enough to influence its expression. In the protandrous gilthead sea bream (*Sparus aurata*) head kidney, *star* expression was not affected by an acute stressor (i.e., net chasing) but *star* mRNA levels increased threefold when a chronic stressor (i.e., overcrowding) was applied [19]. In contrast, an acute stressor (i.e., brief confinement) induced an increase in *star* transcript abundance in the head kidney of rainbow trout [20]. Thus, it appears that responsiveness of *star* to stress may vary between species and depending on the magnitude of the stressor.

Epigenetic reprogramming (e.g., changes in DNA methylation) has been proposed as a key element involved in the regulation of teleost sex change [21]. In the present study, however, genes encoding DNA methylation machinery (*dnmt1* and *dnmt3aa*) were unaffected by the cortisol treatment. These data suggest that an 8–10 days exposure to high cortisol may not be sufficient to affect methylation patterns in the head kidney of spotty wrasses, opposite to the pattern observed for *dnmt3aa* in the gonads of spotty wrasses socially manipulated [22] (Muncaster et al., submitted). It is also possible that methylation at the level of the head kidney is not involved in the orchestration of sex change in general, or that changes in sexually dimorphic DNA methylation are specific to certain species.

# References

1. Frisch A, Walker S, McCormick M, Solomon-Lane TK. Regulation of protogynous sex change by competition between corticosteroids and androgens: an experimental test using sandperch, *Parapercis* *cylindrica*. Hormones and Behavior. 2007;52: 540–545. doi:10.1016/j.yhbeh.2007.07.008

2. Carragher JF, Sumpter JP, Pottinger TG, Pickering AD. The deleterious effects of cortisol implantation on reproductive function in two species of trout, Salmo trutta L. and Salmo gairdneri Richardson. General and Comparative Endocrinology. 1989;76: 310–321. doi:10.1016/0016-6480(89)90163-9

3. Foo JTW, Lam TJ. Serum cortisol response to handling stress and the effect of cortisol implantation on testosterone level in the tilapia, *Oreochromis mossambicus*. Aquaculture. 1993;115: 145–158. doi:10.1016/0044-8486(93)90365-6

4. Maule AG, Schreck CB, Kaattari SL. Changes in the Immune System of Coho Salmon ( *Oncorhynchus kisutch* ) during the Parr-to-Smolt Transformation and after Implantation of Cortisol. Canadian Journal of Fisheries and Aquatic Sciences. 1987;44: 161–166. doi:10.1139/f87-021

5. Pickering AD, Pottinger TG, Carragher JF, Sumpter JP. The effects of acute and chronic stress on the levels of reproductive hormones in the plasma of mature male brown trout, *Salmo trutta* L. General and Comparative Endocrinology. 1987;68: 249–259. doi:10.1016/0016-6480(87)90036-0

6. Vijayan MM, Leatherland JF. Cortisol-induced changes in plasma glucose, protein, and thyroid hormone levels, and liver glycogen content of coho salmon ( *Oncorhynchus kisutch Walbaum* ). Canadian Journal of Zoology. 1989;67: 2746–2750. doi:10.1139/z89-389

7. DiBattista JD, Anisman H, Whitehead M, Gilmour KM. The effects of cortisol administration on social status and brain monoaminergic activity in rainbow trout *Oncorhynchus mykiss*. Journal of Experimental Biology. 2005;208: 2707–2718. doi:10.1242/jeb.01690

8. Pottinger TG, Pickering AD. The effect of cortisol administration on hepatic and plasma estradiol-binding capacity in immature female rainbow trout (*Oncorhynchus mykiss*). General and Comparative Endocrinology. 1990;80: 264–273. doi:10.1016/0016-6480(90)90171-H

9. Richman NH, Tai de Diaz S, Nishioka RS, Prunet P, Bern HA. Osmoregulatory and endocrine relationships with chloride cell morphology and density during smoltification in coho salmon (*Oncorhynchus* *kisutch*). Aquaculture. 1987;60: 265–285. doi:10.1016/0044-8486(87)90293-6

10. Harwood NJ, Lokman PM. Fecundity of banded wrasse (*Notolabrus* *fucicola*) from Otago, Southern New Zealand. New Zealand Journal of Marine and Freshwater Research. 2006;40: 467–476. doi:10.1080/00288330.2006.9517436

11. Lokman PM, Harris B, Kusakabe M, Kime DE, Schulz RW, Adachi S, et al. 11-Oxygenated androgens in female teleosts: prevalence, abundance, and life history implications. General and Comparative Endocrinology. 2002;129: 1–12. doi:10.1016/S0016-6480(02)00562-2

12. Gamperl AK, Vijayan MM, Boutilier RG. Experimental control of stress hormone levels in fishes: techniques and applications. Reviews in Fish Biology and Fisheries. 1994;4: 215–255. doi:10.1007/BF00044129

13. Ramsay JM, Feist GW, Varga ZM, Westerfield M, Kent ML, Schreck CB. Whole-body cortisol response of zebrafish to acute net handling stress. Aquaculture. 2009;297: 157–162. doi:10.1016/j.aquaculture.2009.08.035

14. Barton BA. Stress in fishes: a diversity of responses with particular reference to changes in circulating corticosteroids. Integrative and Comparative Biology. 2002;42: 517–525. doi:10.1093/icb/42.3.517

15. Xie F, Xiao P, Chen D, Xu L, Zhang B. miRDeepFinder: a miRNA analysis tool for deep sequencing of plant small RNAs. Plant Molecular Biology. 2012;80: 75–84. doi:10.1007/s11103-012-9885-2

16. Pfaffl MW, Tichopad A, Prgomet C, Neuvians TP. Determination of stable housekeeping genes, differentially regulated target genes and sample integrity: BestKeeper – Excel-based tool using pair-wise correlations. Biotechnology Letters. 2004;26: 509–515. doi:10.1023/B:BILE.0000019559.84305.47

17. Kruskal WH, Wallis WA. Use of ranks in one-criterion variance analysis. J Am Stat Assoc. 1952;47: 583–621.

18. Miller WL. Steroidogenic acute regulatory protein (StAR), a novel mitochondrial cholesterol transporter. Biochimica et Biophysica Acta (BBA) - Molecular and Cell Biology of Lipids. 2007;1771: 663–676. doi:10.1016/j.bbalip.2007.02.012

19. Castillo J, Castellana B, Acerete L, Planas J V, Goetz FW, Mackenzie S, et al. Stress-induced regulation of steroidogenic acute regulatory protein expression in head kidney of gilthead seabream (*Sparus* *aurata*). Journal of Endocrinology. 2008;196: 313–322. doi:10.1677/JOE-07-0440

20. Kusakabe M, Todo T, McQuillan HJ, Goetz FW, Young G. Characterization and expression of steroidogenic acute regulatory protein and MLN64 cDNAs in trout. Endocrinology. 2002;143: 2062–2070. doi:10.1210/endo.143.6.8672

21. Todd E V, Ortega-Recalde O, Liu H, Lamm MS, Rutherford KM, Cross H, et al. Stress, novel sex genes, and epigenetic reprogramming orchestrate socially controlled sex change. Science Advances. 2019;5: eaaw7006. doi:10.1126/sciadv.aaw7006

22. Goikoetxea A. Stress and sex change in New Zealand spotty wrasse (Notolabrus celidotus). University of Otago. 2020. Available: http://hdl.handle.net/10523/9946

# Supplementary Figures


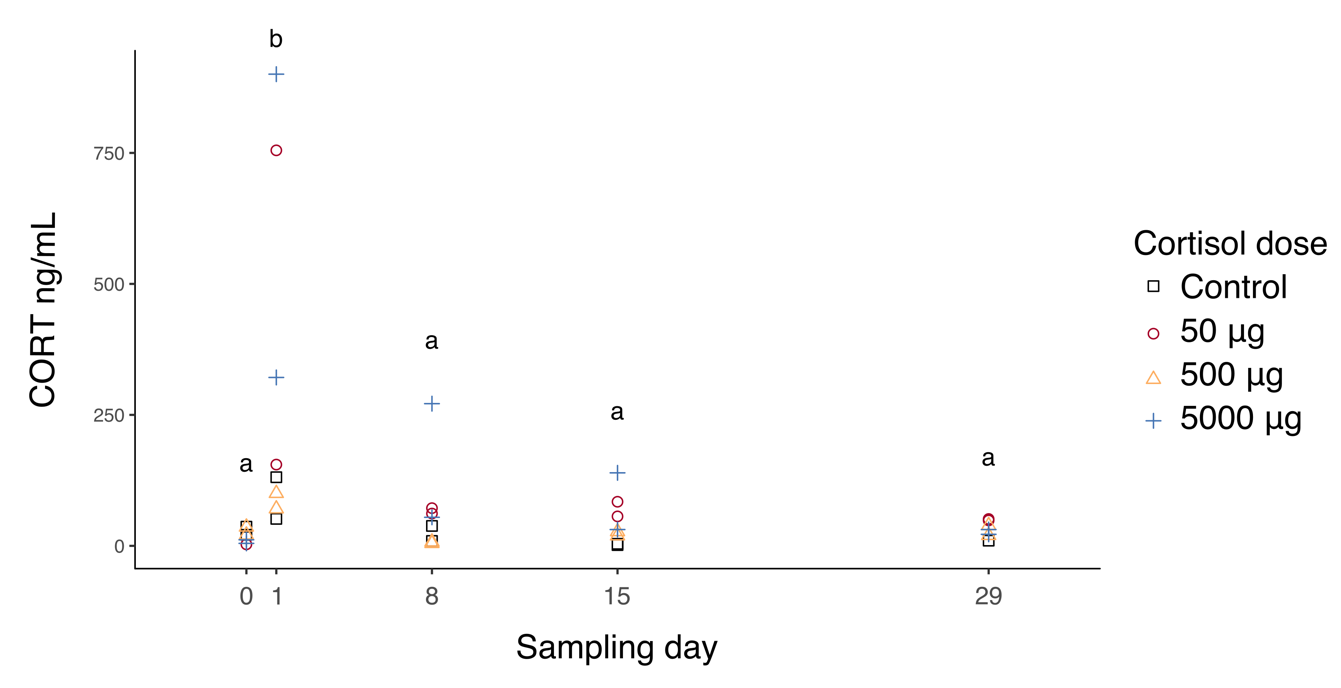


**Supplementary Figure 1.** Radioimmunoassay measurement of plasma levels of cortisol (CORT) in fish implanted with 0 (control), 50, 500 and 5000 μg cortisol pellets, respectively. Letters denote a significant difference in distribution between groups.


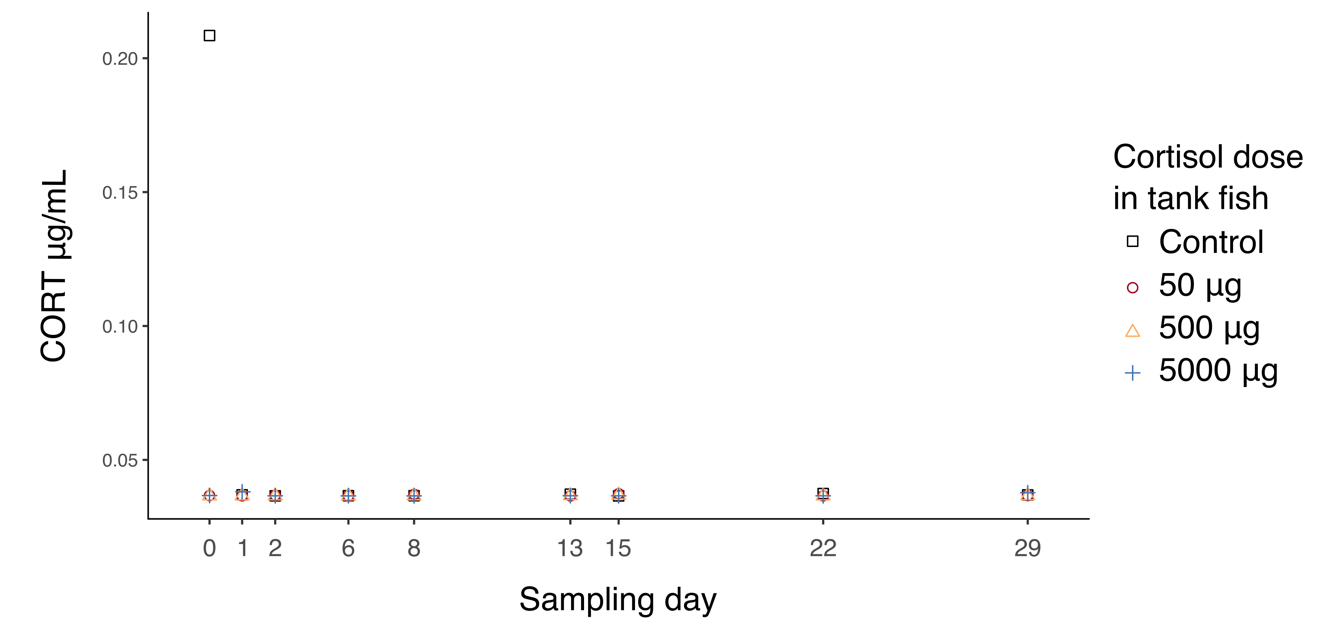


**Supplementary Figure 2.** HPLC measurement of cortisol (CORT) levels in the water from tanks containing fish implanted with control (0 μg) or cortisol pellets (50, 500 and 5000 μg; respectively).

**
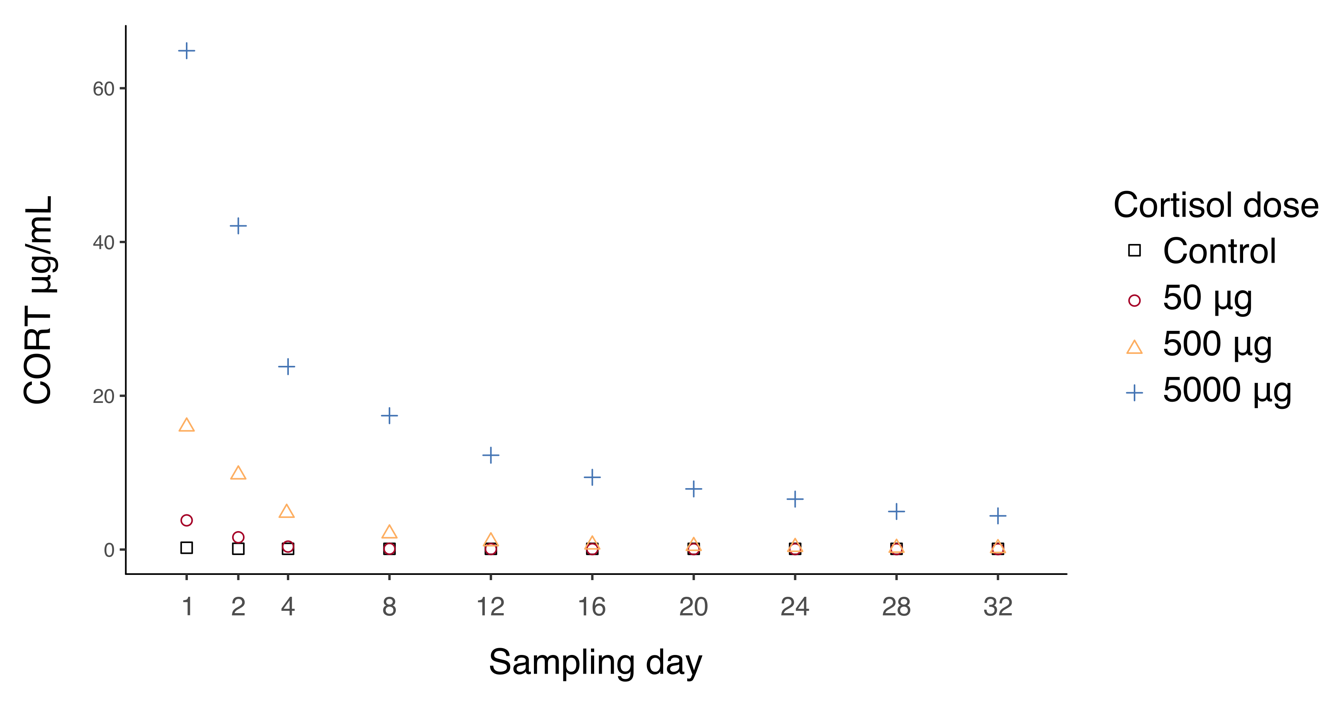
**

**Supplementary Figure 3.** HPLC measurement of mean cortisol (CORT) levels in salmon Ringer’s solution in contact with cortisol pellets (50, 500 and 5000 μg; respectively).

**Supplementary Table 1.** Overview of potential reference genes from gonadal samples ranked from higher to lower stability (top to bottom) using different statistical approaches. RefFinder, Δ CT, BestKeeper SD and NormFinder rankings were obtained in RefFinder (A), while BestKeeper r was calculated on excel-based BestKeeper (B). Abbreviations: average (Ave.), geometric mean (GM), Pearson’s correlation coefficient (r), standard deviation (SD), stability value (SV).

(A)

| RefFinder | | Δ CT | | BestKeeper | | | | NormFinder | |
| --- | --- | --- | --- | --- | --- | --- | --- | --- | --- |
| Genes | GM | Genes | Ave. SD | Genes | SD | Genes | r | Genes | SV |
| *actb1* | 1.19 | *actb1* | 20428.86 | *g6pd* | 297.75 | *g6pd* | 0.68 | *actb1* | 6928.25 |
| *g6pd* | 1.59 | *g6pd* | 26422.94 | *actb1* | 10522.29 | *actb1* | 0.91 | *g6pd* | 22171.02 |
| *eef1a1a* | 3.00 | *eef1a1a* | 32995.31 | *eef1a1a* | 30432.09 | *eef1a1a* | 0.93 | *eef1a1a* | 32072.08 |

(B)

172

| BestKeeper – Excel-based tool | | | | |
| --- | --- | --- | --- | --- |
| Genes | SD | r | r^2^ | p-value |
| *g6pd* | 297.75 | 0.68 | 0.46 | 0.001 |
| *actb1* | 10522.29 | 0.91 | 0.84 | 0.001 |
| *eef1a1a* | 30432.09 | 0.93 | 0.86 | 0.001 |


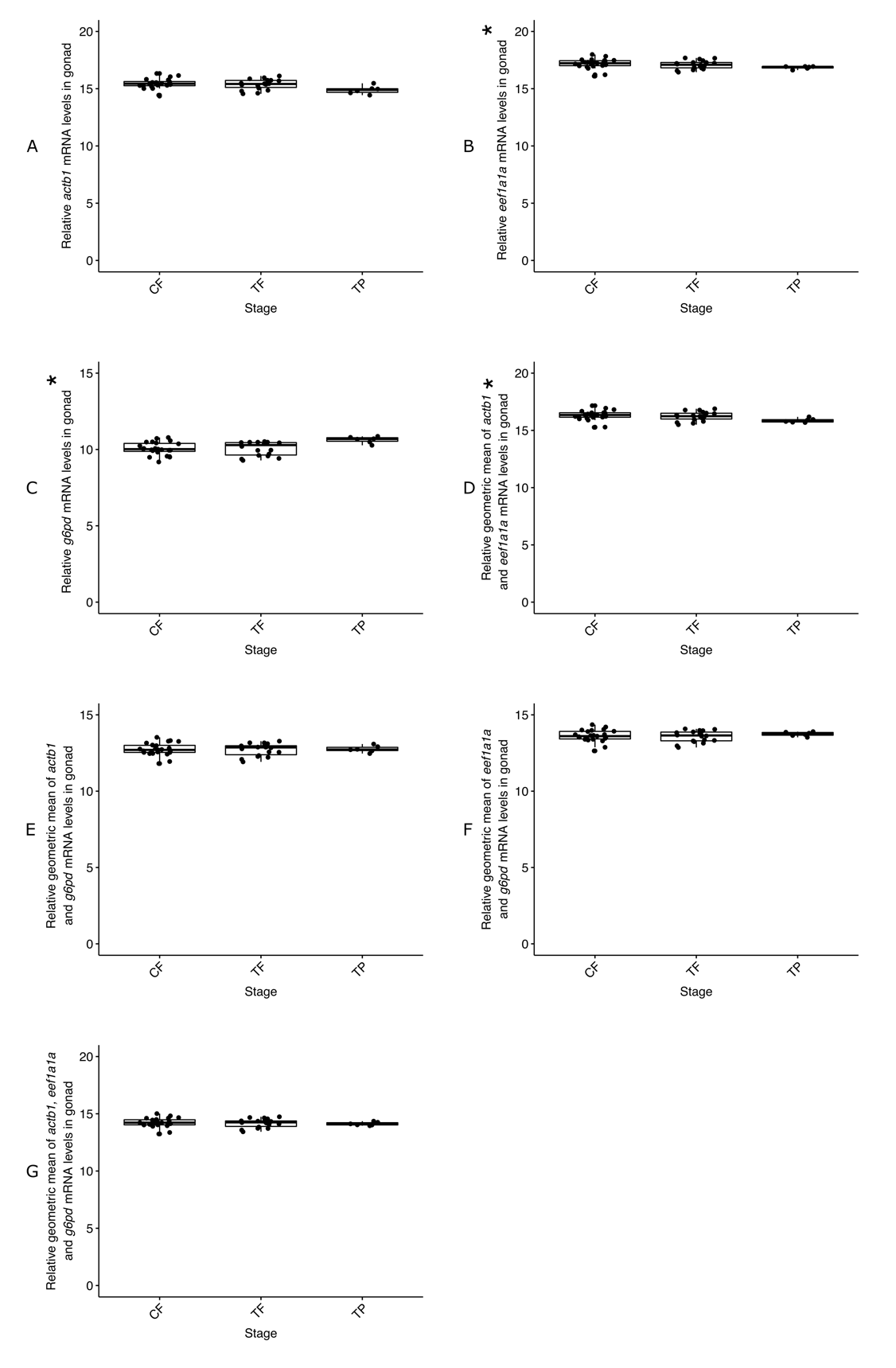


**Supplementary Figure 4.** Relative mRNA levels of each candidate reference gene, *actb1* (A), *eef1a1a* (B), *g6pd* (C); geometric mean of *actb1* and *eef1a1a* (D), geometric mean of *actb1* and *g6pd* (E), geometric mean of *eef1a1a* and *g6pd* (F), and geometric mean of *actb1*, *eef1a1a* and *g6pd* (G) in the gonad. * indicates a significant effect of treatment on relative mRNA levels. Sample sizes: CF^☨^ n = 23, TF n = 18, TP n = 6. Abbreviations: control female (CF), treatment female (TF), control terminal phase male (TP). ^☨^Both control females sampled on day 0 of the experiment (n = 5), and females used as controls throughout the experiment (n = 18) were grouped altogether as CF for the purpose of this analysis.

**Supplementary Table 2.** Overview of potential reference genes from head kidney samples ranked from higher to lower stability (top to bottom) using different statistical approaches. RefFinder, Δ CT, BestKeeper SD and NormFinder rankings were obtained in RefFinder (A), while BestKeeper r was calculated on excel-based BestKeeper (B). Abbreviations: average (Ave.), geometric mean (GM), Pearson’s correlation coefficient (r), standard deviation (SD), stability value (SV).

(A)

178

| RefFinder | | Δ CT | | BestKeeper | | | | NormFinder | |
| --- | --- | --- | --- | --- | --- | --- | --- | --- | --- |
| Genes | GM | Genes | Ave. SD | Genes | SD | Genes | r | Genes | SV |
| *g6pd* | 1.26 | *g6pd* | 12777.84 | *l36* | 8.97 | *l36* | 0.73 | *g6pd* | 522.62 |
| *l36* | 1.59 | *l36* | 13191.56 | *g6pd* | 814.308 | *g6pd* | 0.76 | *l36* | 4604.04 |
| *actb1* | 2.28 | *actb1* | 24924.16 | *actb1* | 20890.76 | *actb1* | 0.86 | *actb1* | 24916.63 |

(B)

| BestKeeper – Excel-based tool | | | | |
| --- | --- | --- | --- | --- |
| Genes | SD | r | r^2^ | p-value |
| *l36* | 8.97 | 0.73 | 0.53 | 0.001 |
| *g6pd* | 814.308 | 0.76 | 0.58 | 0.001 |
| *actb1* | 20890.76 | 0.86 | 0.73 | 0.001 |


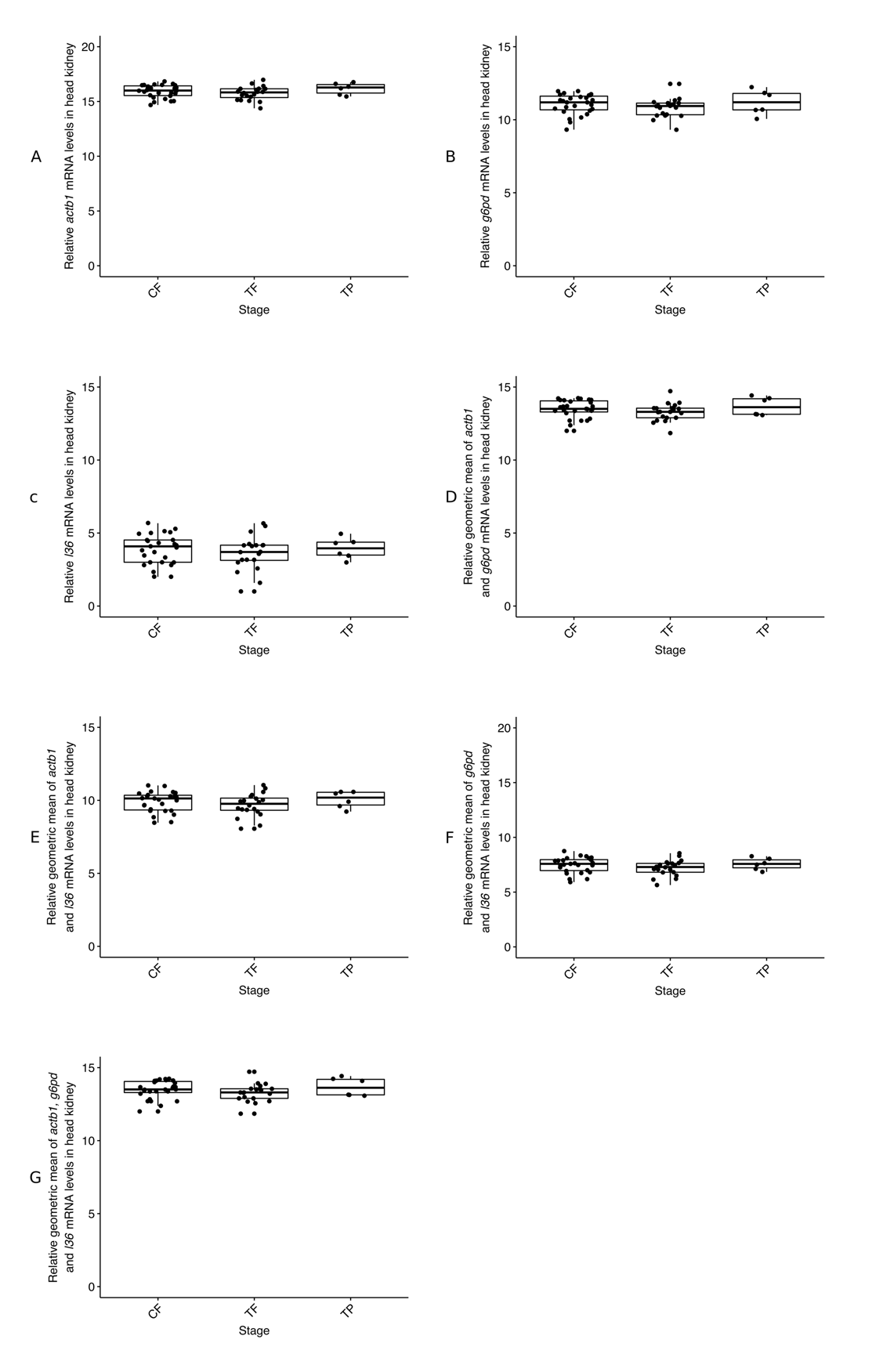


**Supplementary Figure 5.** Relative mRNA levels of each candidate reference gene, *actb1* (A), *g6pd* (B), *l36* (C); geometric mean of *actb1* and *g6pd* (D), geometric mean of *actb1* and *l36* (E), geometric mean of *g6pd* and *l36* (F), and geometric mean of *actb1*, *g6pd* and *l36* (G) in the head kidney. Sample sizes: CF^☨^ n = 27, TF n = 20, TP n = 6. Abbreviations: control female (CF), treatment female (TF), control terminal phase male (TP). ^☨^Both control females sampled on day 0 of the experiment (n = 6), and females used as controls throughout the experiment (n = 21) were grouped altogether as CF for the purpose of this analysis.

**Supplementary Table 3.** Expanded suite of 19 candidate genes analysed in spotty wrasse gonad using the nanoString nCounter^TM^ CodeSet technology. Abbreviations: high mobility group (HMG), sex-determining region (SRY).

| Gene Symbol | Gene Description | Contig ID | Reference transcript ID |
| --- | --- | --- | --- |
| Housekeeping genes | | | |

| *actb1* | β-actin, cytoplasmic 1 | c58053_g1_i1 | NM_131031.1 |
| --- | --- | --- | --- |
| *eef1a1a* | eukaryotic translation elongation factor 1 alpha 1a | c58053_g1_i1 | NM_200009.2 |
| *g6pd* | glucose-6-phosphate dehydrogenase | c39960_g1_i1 | ENSDART00000104834.6 |

| Steroidogenesis and hormone receptors | | | |
| --- | --- | --- | --- |
| *cyp19a1a* | aromatase a (gonad isoform) | c52027_g1_i1 | NM_131154.3 |
| *cyp11c1/b2* | steroid 11β-hydroxylase | c62027_g1_i1 | NM_001080204.1 |
| *nr3c1* | glucocorticoid receptor | c36910_g2_i1 | NM_001020711.3 |
| *nr3c2* | mineralocorticoid receptor | c49976_g1_i2 | NM_001100403.1 |
| Key sex-related transcription factors | | | |
| *foxl2a* | forkhead box L2a | c53356_g1_i1 | NM_001045252.2 |
| *dmrt1* | doublesex and mab-3 related transcription factor 1 | c66498_g1_i1 | NM_205628.2 |
| *amh* | anti-Müllerian hormone | c51546_g1_i1 | NM_001007779.1 |
| *sox9a* | SRY-related HMG box 9a | c53707_g2_i1 | NM_131643.1 |
| Rspo1/Wnt/β-catenin pathway | | | |
| *ctnnb1* | catenin (cadherin-associated protein), beta 1 | c47984_g1_i2 | NM_131059.2 |
| E3 ubiquitin-protein ligase | | | |
| *znrf3* | zinc and ring finger 3 | c68386_g1_i1 | NM_001308555.1 |
| *fancl* | Fanconi anaemia complementation group L | c63372_g2_i1 | NM_212982.1 |
| Epigenetic regulatory factors | | | |
| *dnmt1* | DNA methyltransferase 1 | c43163_g1_i1 | NM_131189.2 |
| *dnmt3aa* | DNA methyltransferase 3aa | c59097_g4_i2 | NM_001018134.1 |
| Jumonji gene family | | | |
| *jarid2b* | jumonji, AT rich interactive domain 2b | c67175_g1_i2 | NM_001202459.1 |
| *kdm6bb* | lysine (K)-specific demethylase 6B, b | c52506_g1_i1 | NM_001030178.2 |
| Pluripotency factor | | | |
| *pou5f3* | POU domain, class 5, transcription factor 1 | c63041_g1_i1 | NM_131112.1 |


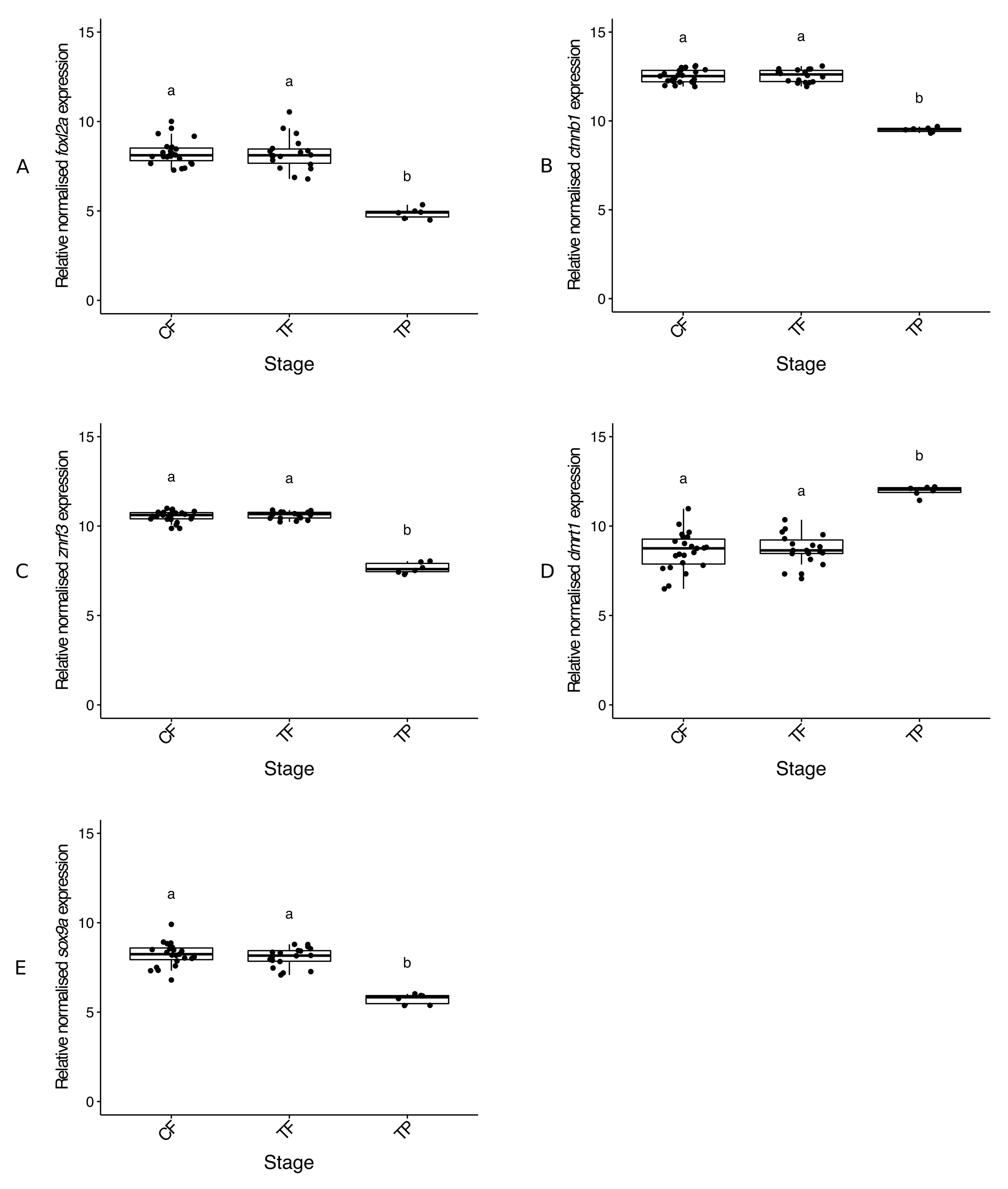


**Supplementary Figure 6**. Relative gonadal expression of *foxl2a* (A), *ctnnb1* (B), *znrf3* (C), *dmrt1* (D) and *sox9a* (E) mRNA. Expression levels are compared among control females, cortisol-implanted females and TP males. In the boxplots, each point represents an individual fish, the middle bold line represents the median, the edges of the box represent the upper and lower quartiles, and vertical lines represent the minimum and maximum values. Letters denote a significant difference in distribution between groups. Sample sizes: CF^☨^ n = 23, TF n = 18, TP n = 6. Abbreviations: control female (CF), treatment female (TF), control terminal phase male (TP). ^☨^Both control females sampled on day 0 of the experiment (n = 5), and females used as controls throughout the experiment (n = 18) were grouped altogether as CF for the purpose of this analysis.


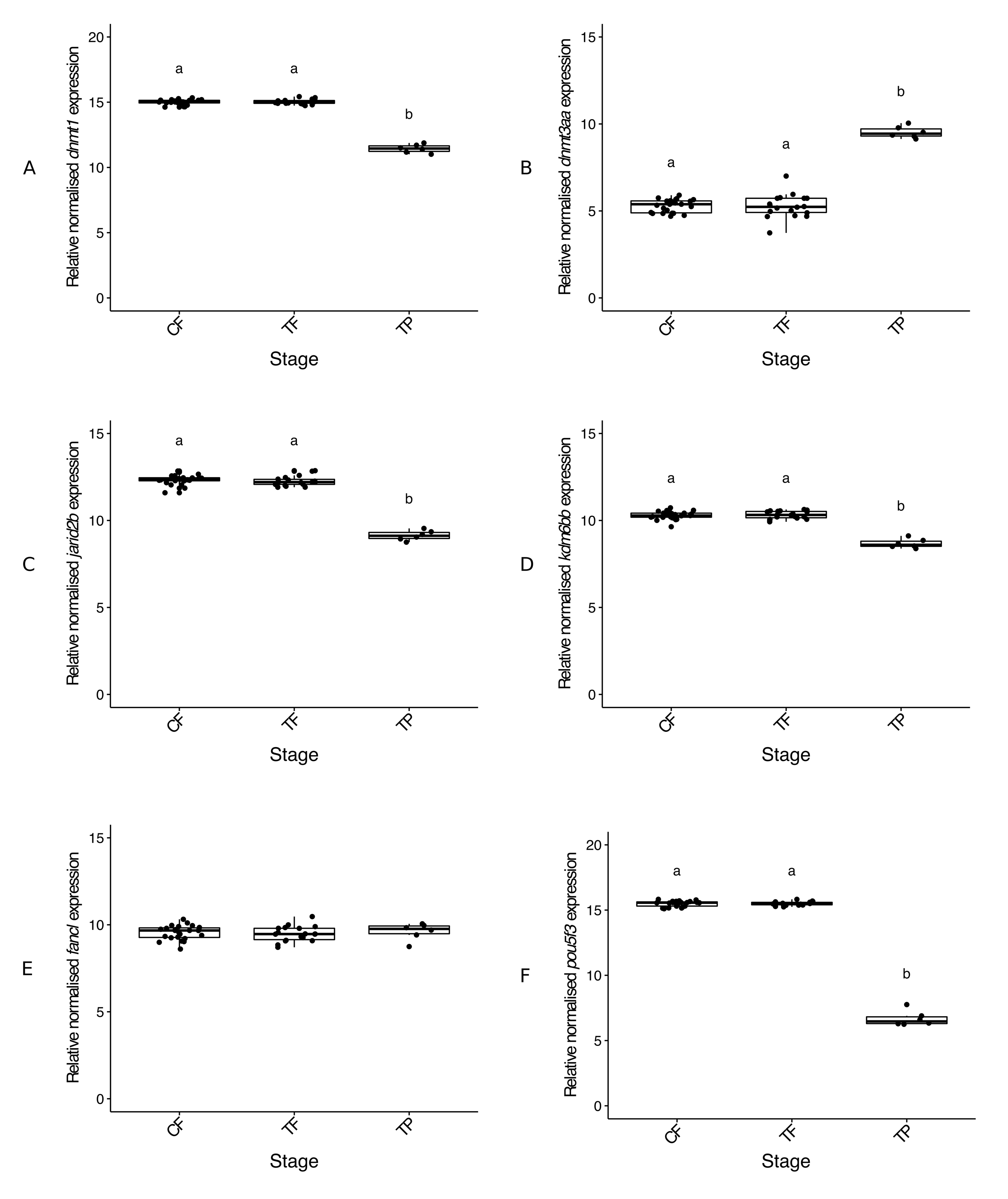


**Supplementary Figure 7**. Relative gonadal expression of *dnmt1* (A), *dnmt3aa* (B), *jarid2b* (C), *kdm6bb* (D)*, fancl* (E) and *pou5f3* (F) mRNA. Expression levels are compared among control females, cortisol-implanted females and TP males. In the boxplots, each point represents an individual fish, the middle bold line represents the median, the edges of the box represent the upper and lower quartiles, and vertical lines represent the minimum and maximum values. Letters denote a significant difference in distribution between groups. Sample sizes: CF^☨^ n = 23, TF n = 18, TP n = 6. Abbreviations: control female (CF), treatment female (TF), control terminal phase male (TP). ^☨^Both control females sampled on day 0 of the experiment (n = 5), and females used as controls throughout the experiment (n = 18) were grouped altogether as CF for the purpose of this analysis.

**
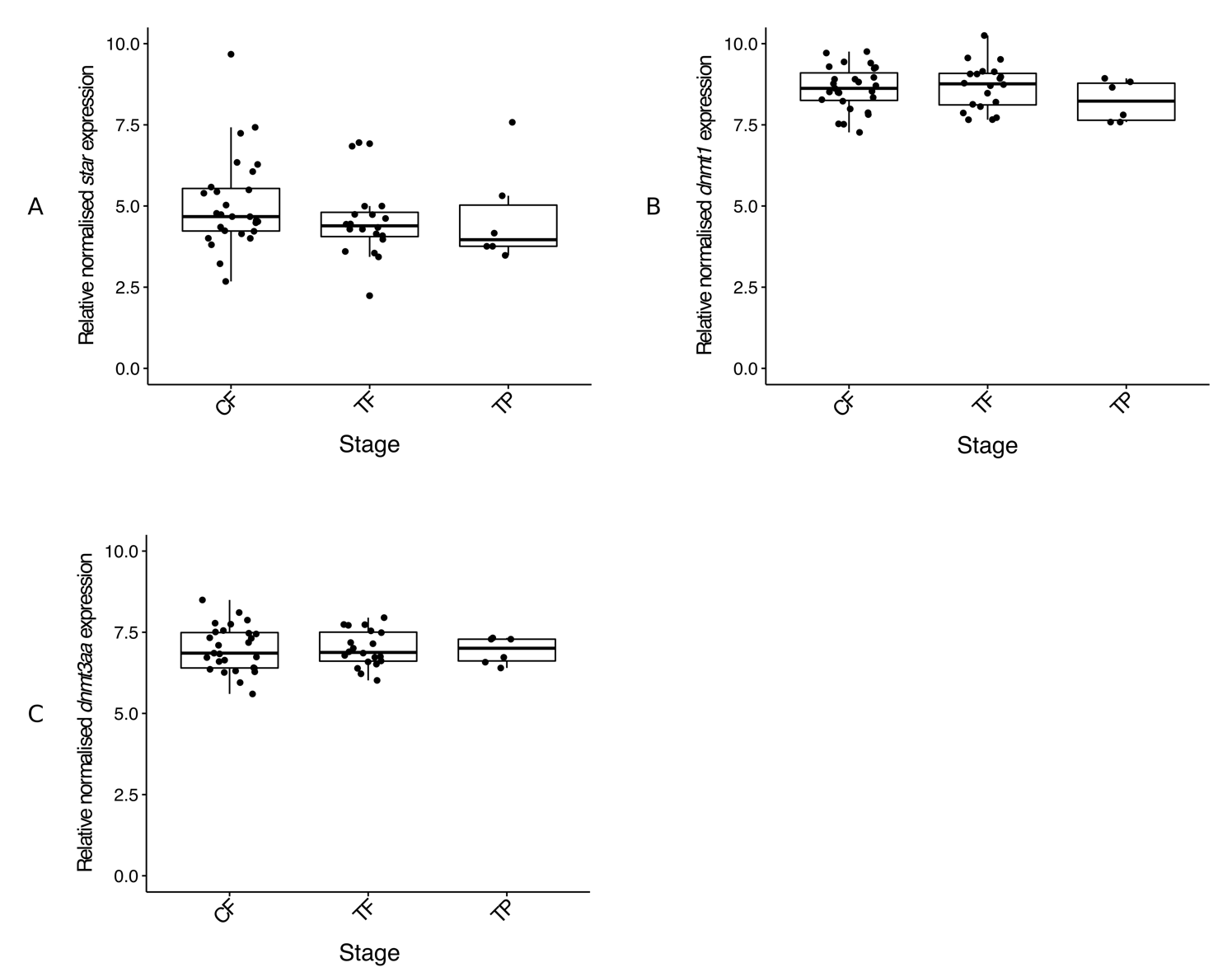
**

**Supplementary Figure 8.** Relative head kidney expression of *star* (A), *dnmt1* (B) and *dnmt3aa* (C) mRNA. Expression levels are compared among control females, cortisol-implanted females and TP males. In the boxplots, each point represents an individual fish, the middle bold line represents the median, the edges of the box represent the upper and lower quartiles, and vertical lines represent the minimum and maximum values. Sample sizes: CF n = 27, TF n = 20, TP n = 6. Abbreviations: control female (CF), treatment female (TF), control terminal phase male (TP).
